# Computational Modeling of a Transcriptional Switch Underlying B-Lymphocyte Lineage Commitment of Hematopoietic Multipotent Cells

**DOI:** 10.1371/journal.pone.0132208

**Published:** 2015-07-13

**Authors:** Luca Salerno, Carlo Cosentino, Giovanni Morrone, Francesco Amato

**Affiliations:** 1 Laboratory of Biomechatronics, Department of Experimental and Clinical Medicine, University Magna Græcia of Catanzaro, Catanzaro, Italy; 2 Laboratory of Molecular Haematopoiesis and Stem Cell Biology, Department of Experimental and Clinical Medicine, University Magna Græcia of Catanzaro, Catanzaro, Italy; University of Southampton, UNITED KINGDOM

## Abstract

Despite progresses in identifying the cellular mechanisms at the basis of the differentiation of hematopoietic stem/progenitor cells, little is known about the regulatory circuitry at the basis of lineage commitment of hematopoietic multipotent progenitors. To address this issue, we propose a computational approach to give further insights in the comprehension of this genetic mechanism. Differently from T lymphopoiesis, however, there is at present no mathematical model describing lineage restriction of multipotent progenitors to early B-cell precursors. Here, we provide a first model—constructed on the basis of current experimental evidence from literature and of publicly available microarray datasets—of the genetic regulatory network driving the cellular fate determination at the stage of lymphoid lineage commitment, with particular regard to the multipotent-B-cell progenitor transition. By applying multistability analysis methods, we are able to assess the capability of the model to capture the experimentally observed switch-like commitment behavior. These methods allow us to confirm the central role of zinc finger protein 521 (ZNF521) in this process, that we had previously reported, and to identify a novel putative functional interaction for *ZNF521*, which is essential to realize such characteristic behavior. Moreover, using the devised model, we are able to rigorously analyze the mechanisms underpinning irreversibility of the physiological commitment step and to devise a possible reprogramming strategy, based on the combined modification of the expression of *ZNF521* and *EBF1*.

## Introduction

The differentiation of hematopoietic cell is governed by several control mechanisms, at different levels and in a highly hierarchical manner, from hematopoietic stem cells (HSCs) to lymphoid multipotent progenitors (LMPPs) that can further develop toward lineage restricted progenitors (LRPs). In these mechanisms, some transcription factors play a key role in the determination of the hematopoietic lineage. Observing these mechanisms from a systemic viewpoint, the following questions arise: *(i)* Which are the upstream regulators of the transcription factors that determine the lineage of differentiation of a certain progenitor or immature cell? *(ii)* Which are the transcription factors that constitute a minimal set for the activation of the differentiation process in hematopoietic cells? *(iii)* Is the differentiation an irreversible event (i.e., once the cell is committed to a certain path, it cannot change), or is it possible to observe phenomena of plasticity? While these and other issues, related to the regulation of hematopoietic development and differentiation, clearly require extensive and time-consuming experimental investigations, the exploitation of mathematical models and associated theoretical analysis tools can significantly boost the comprehension of the lineage specification mechanisms.

The control of HSCs differentiation towards mature hematopoietic cells is a sequential process, consisting of several lineage-determining steps. Each step requires a decision mechanism, which is assumed to be highly regulated by a combination of transcription factors, epigenetic events, and extrinsic regulator cytokines [1]. Prior to commitment, it has been observed that many genes involved in HSCs differentiation are expressed at intermediate or basal levels [2, 3]. This “transcriptional priming” at the stage of early progenitors may induce the rapid deployment of transcription factors to implement the subsequent commitment mechanisms. In hematopoiesis there exist several lineage branch points with identified key transcription factors and external signals [4–6]. For instance, a particularly well studied subnetwork is the one involving the genes *PU.1* and *GATA-1*, which underlies the erythroid-myeloid lineage-determination step and has been proved to exhibit both a commitment switch and priming features [7–12]. In many cases, switch-like gene circuits have evolved to realize a sort of cellular built-in memory, which can lead to phenotypic diversity [13].

Several models have been proposed to describe a late stage of B-lymphocytes differentiation into terminal B-cells: sustained exposure to CD40L has been suggested to direct germinal center B-cells toward the memory B-cell compartment [14]; a bistable switch arising from the coupled double-negative feedback loops involving BCL-6, BLIMP-1 and PAX5 forms the basis of the B-cell to plasma cell differentiation program and its disruption by dioxin [15]; in [16], the authors developed a kinetic model to quantitatively characterize B-cell exit from the germinal center phase and terminal differentiation into plasma and memory B-cells.

Differently from those related to the terminal phases, the regulatory mechanisms underlying other lineage-determination steps are still poorly understood: for instance, to our knowledge, no model of the early B-lymphopoiesis commitment switch has been devised so far. Thus, the present work focuses on the analysis of this important mechanism. In particular, we first develop a dynamical model in order to clearly identify a regulatory network of genes/transcription factors involved in cellular fate determination of B-lymphoid lineage specification, based on the experimental evidence reported in literature. A special emphasis is given to the role of ZNF521, a key regulator and crucial antagonist of some genes involved in the B-lymphoid commitment regulatory network, as recently proved in experimental studies [17, 18]. Morrone and coworkers have suggested that ZNF521, in concert with other transcription factors, influences self-renewal and differentiation of primitive progenitors in hematopoiesis.

Exploiting the proposed mathematical model, we have studied the gene network regulating B-lymphoid commitment switch; in particular, we have investigated the role of several novel putative interactions by comparing the behavior of the alternative models against the available experimental data. Bifurcation analysis and numerical simulations have shown that the currently known interactions are not sufficient to generate the bistable behavior that distinguishes biological switches; moreover, the analysis of the extended models quantitatively proves that a novel interaction, that is the inhibition of *ZNF521* expression by the B-cell lineage specific activator protein PAX5 [19], is required for the model to exhibit the commitment switch. Finally, the devised mathematical model is exploited to investigate possible strategies for reprogramming B-lymphoid LRPs into LMPPs.

## Results

### B-cell lineage commitment model and the role of ZNF521

**Fig 1:**
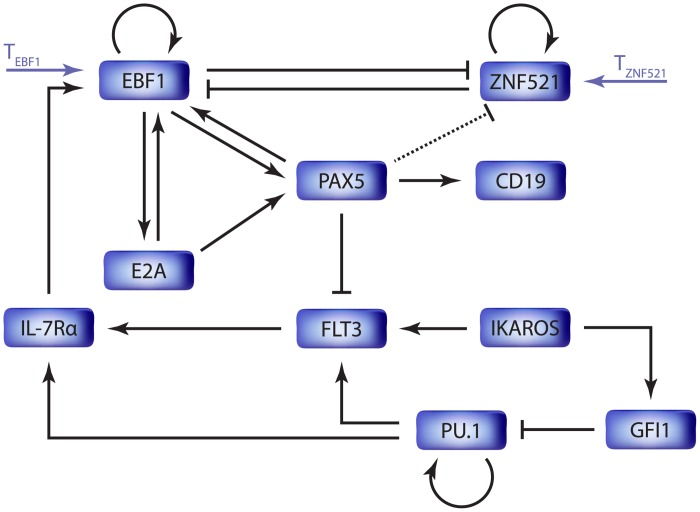
The key TF interactions in the B-lymphopoiesis circuit. The B-cell lineage is assumed to be determined by the antagonism between ZNF521 and the specification of the B-lymphoid lineage driven by E2A, EBF1 and PAX5. The interaction framework emerge out of literature. It is also supported by some assumptions from the phenomenological observation that can lead to cellular switching. In order to gain this particular behavior, considering well-known transcriptional motifs, we assumed a putative direct inhibition of ZNF521 through the regulator PAX5, (dashed line). Arrows represent gene activation, blunt arrows gene repression. *T*_*EBF*1_ and *T*_*ZNF*521_, indicate environmental factors, which promote the transcriptional activity of EBF1 and ZNF521, respectively.

Robust cell fate commitment is based on the activation and inhibition of the expression of certain genes. The two cytokine receptors, FLT3 and IL-7R, and the seven transcription factors PU.1, IKAROS, GFI1, E2A, EBF1, PAX5 and ZNF521 are critical for the development of B-cell precursors. These factors form a gene regulatory network which activates during LMPPs commitment towards B-lymphocytes development. We have assembled the functional interaction network, shown in Fig 1, by manually mining the information from the literature, as reported in S1 Text.

From a dynamical system perspective, this biological network implements a switch-like function and, therefore, is expected to exhibit a bistable behavior, i.e., it can robustly operate in two distinct conditions. To enable the application of the analysis tools for bistability, we have complemented the qualitative description provided by the network in Fig 1 with kinetic rules associated to each interaction, thus devising the dynamical model Eqs (1a)–(1j). The model implicitly assumes that the activity levels of transcription factors and cytokine receptors are roughly proportional to their mRNA levels. In order to analyze their effect on early B-lymphopoiesis lineage commitment the model includes two environmental factors, *T*_*EBF*1_ and *T*_*ZNF*521_, which promote the transcriptional activity of *EBF1* and *ZNF521*, respectively. Modulating the effect of these exogenous inputs, we can induce the switch between the two stable operative conditions, analyzing the effect of EBF1 and ZNF521 on a continuous range of expression levels.

**Fig 2:**
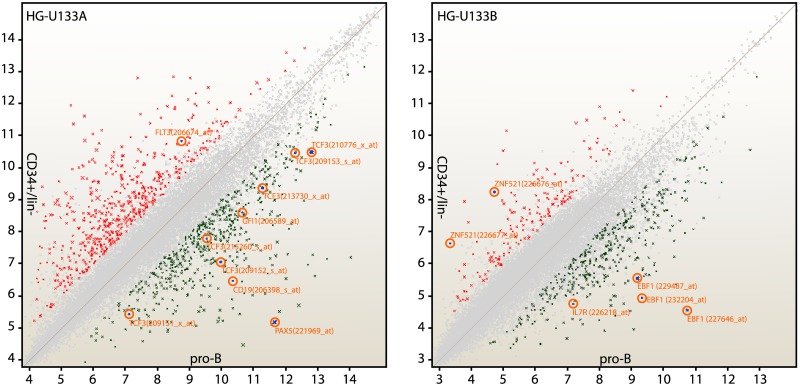
Gene level differential expression analysis. Every point in the scatter plots shows the expression of a gene in the two conditions CD34^+^/lin^−^ vs. pro-B. **HG-U133A** Chip. Total number of genes evaluated: 22645, with 550 genes differentially expressed. CD34^+^/lin^−^ vs. pro-B analysis shows that 167 genes are up-regulated and 383 genes are down-regulated. **HG-U133B** Chip. Total number of genes evaluated: 22283 with 1080 genes differentially expressed CD34^+^/lin^−^ vs. pro-B analysis shows that 553 genes are up-regulated and 527 genes are down-regulated. According with the TAC default filter criteria, we setted a fold change (linear) cut-off of ±2 (all that points having a fold-change less than 2 are shown in gray) and p-value < 0,05. Red: up in CD34^+^/lin^−^ vs pro-B; green: down in CD34^+^/lin^−^ vs pro-B. Highlighted are shown the factors involved in the proposed model.

The dynamics of the system cannot be determined without first specifying the parameter values, fixing a point in the parameter space. The qualitative behavior of the commitment switch in the B lymphopoiesis lineage is adapted, firstly, by a set of biochemical parameters for the system in Eqs (1a)–(1j) that is reasonably consistent with the expression levels of microarray gene profiles of the CD19^+^ CD127^+^ and CD19^+^ CD127^−^ cells with B lineage cell subsets isolated from pediatric marrow reported in [20, 21]. Since these earlier studies have been conducted using the HG-U133 chip set (HG-U133A and HG-U133B), analysis has been conducted over the 44,754 probe sets present in the two array types, which were then processed using the Affymetrix platform Expression Console. The application uses the Robust Multichip Analysis (RMA) in order to obtain the generation of experimental expression datasets. Further statistical analysis is performed considering Affymetrix Transcriptome Analysis Console (TAC) Software. Considering the different developmental lineages, we obtained a list of differentially expressed genes in genome-wide comparison between multipotent stage and B lymphoid LRPs (pro-B), as reported in Fig 2 and S1 Fig.

### Cell lineage determination in the state space

The state of our dynamical system, let us denote it by *X*, is defined by the expression levels of the genes included in the regulatory network in Fig 1, that is *X* = (*x*_ikaros_, *x*_gfi1_, *x*_pu.1_, …, *x*_cd19_), where *x*_ikaros_ denotes the concentration of transcribed IKAROS mRNA. A point in the state space is a stable equilibrium if every trajectory of the system starting in a small neighborhood of that point remains close to it for an infinite time interval in the absence of exogenous inputs.

In order to implement a switch-like behavior, our system should exhibit two distinct equilibrium points: some genes will have high expression in one point and low in the other one, while other genes may settle on an intermediate expression level in both conditions. These two equilibrium points of our model represent the multipotent progenitors and committed B precursors conditions, and the model trajectories are attracted on one or the other depending on the initial condition [22]. In general, all the state trajectories are limited by gene regulatory interactions that represent effectively constraints in the dynamic evolution [23]. Indeed cellular development creates only a small subset of states among all the possible states because a cell state does not move entirely randomly. Obviously, individual genes do not alter their expression value independently because of predetermined regulatory interactions. No gene regulatory interactions entails free movement of states. In the proposed model, the admissible state trajectories are characterized by implicit constraints: for instance, if the transcription factor ZNF521 inhibits the expression of *EBF1*, then as *ZNF521* increases its expression EBF1 is subjected to decrease.

### A key circuit promotes irreversible bistable commitment switch between LMPPs and lymphoid LRPs

In correspondence to the lymphoid multipotent stage, the two transcription factors IKAROS and PU.1 initialize the lymphoid lineage commitment process, in particular activating the transcription of FLT3, a receptor specifically expressed on the surface of LMPPs [24, 25]. PU.1, in turn, is maintained at lower levels for the cell to persist in lymphoid line, avoiding the switching to the myeloid one. GFI1 (activated by IKAROS) has been suggested to interact in a direct manner with the promoter of *SPI1* (the gene encoding PU.1 transcription), inhibiting its expression; hence, IKAROS indirectly lowers PU.1 level [26]. Our analysis has highlighted that the expression level of PU.1 cannot be simply nullified, but it must be finely regulated: indeed, its presence is necessary for the regulation of the expression of IL-7R, which, in turn, is required for the progression of the development from LMPPs to B-lymphoid lineage [27, 28].

**Fig 3:**
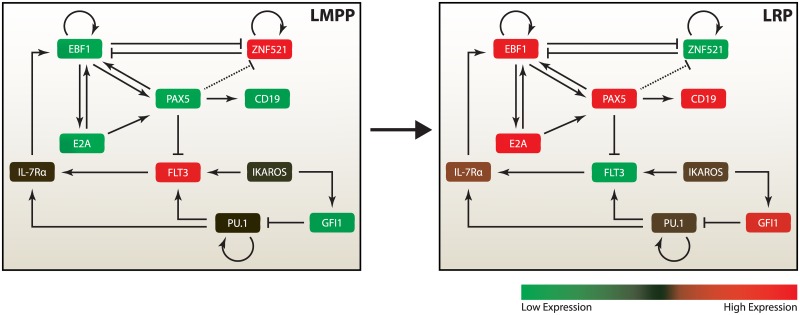
Expression programs characterizing the commitment of LMPPs toward B-lymphoid LRPs. Each state is guaranteed by a distinctive expression program of regulatory factors. In red (green), are depicted the factors up (down) regulated in each condition, LMPP and LRP, respectively. Values are taken in arbitrary units from the experimental gene profiling datasets, reported in [20].

The lymphoid LRPs commitment is guaranteed by the antagonism between ZNF521 and the B-lymphoid lineage specific genes, like *TCF3* (coding for E2A trascription factor), *EBF1* and *PAX5*, see Fig 3. While most of the interactions between these factors have been unraveled by means of biological experiments, evidence of regulation due to higher order multimeric bindings is lacking; therefore, in our model we admit only heterodimeric pairwise. If this modeling assumption was removed, one could rather easily (and arbitrarily) tailor high-order kinetics to obtain bistable dynamics. Our approach, instead, aims at the discovery of the key interactions underpinning the lineage commitment switch and are, therefore, the most promising candidates to be investigated via wet-lab experiments.

The model built on the basis of the currently known experimentally validated interactions is not able to reproduce the commitment switch behavior, i.e. it never achieves bistability, independently of the assigned parameter values. This suggests that part of the regulatory network still ought to be unveiled. Consequently, we have exploited the devised model to test additional hypothetical interactions, chosen among the most likely on the basis of the literature and of the knowledge of the domain expert.

The proposed *in silico* model, thus, provides an effective tool to rapidly sieve multiple hypotheses on the topology of the interaction network. From the examination of different network structures, only one interaction, namely the direct inhibition of the regulator PAX5 on *ZNF521*, has resulted to be crucial to reproduce the dynamics of a bistable lineage commitment switch. In fact, removing this inhibitory action of PAX5 on *ZNF521* from the model, the system does no longer exhibit a switching characteristic, even in the presence of other additional interactions. For example, an alternative model featuring inhibition of ZNF521 through E2A cannot yield bistable behavior, see Model B in S1 Text and S6 Fig. Recently, mouse studies on the transcription factor Ikaros in early B-cell development have confirmed its role of regulator in lymphopoiesis, revealing also different activated or repressed target genes, among which the murine equivalent for ZNF521 [29]. Based on these experimental evidences, a further alternative model characterized by a direct repression on *ZNF521* through IKAROS has been considered, Model C in S1 Text and S6 Fig. However, numerical simulations suggest that repression of IKAROS on *ZNF521* is not able to induce bistable switching, see S6 Fig. Several other alternative interactions have been tested during the construction of the model, confirming that the PAX5/ZNF521 interaction is essential to the realization of the switching mechanism.

**Fig 4:**
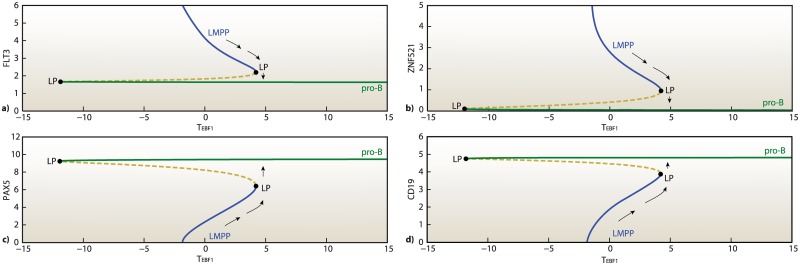
Irreversible and bistable switch in LMPPs commitment into B-lymphoid LRPs (pro-B cells). Steady state concentrations of FLT3, ZNF521, PAX5 and CD19 as functions of an external signal *T*_*EBF*1_, which promotes B-cell development and represses ZNF521. All the points located on the negative semiaxis, the shaded area, are inaccessible (expression levels of some species assume negative values). LP, Limit Point.

The mechanism linking EBF1 expression level to B-lymphoid LRPs commitment has the characteristics of an irreversible bistable switch, mediated by the feedback between ZNF521-EBF1, PAX5, and the multipotency marker FLT3. The system behavior changes are evaluated versus variations in control parameter regulating the transcriptional activity of EBF1 expression, *T*_*EBF*1_, by means of bifurcation analysis [30]. Externally modifying the value of *T*_*EBF*1_, it is possible to replicate experimental variation in the expression of EBF1, essential for the commitment of B-lymphocyte identity. Fig 4 shows a typical bistable characteristic, featuring the presence of two stable steady-state branches delimited by two bifurcation points. All the points included in the region of bistability admit either a low or high value in correspondence to a single value of the bifurcation parameter. Note also that the bifurcation point located on the negative semiaxis is inaccessible (expression levels cannot achieve negative values), therefore the two branches of the bifurcation diagram are disconnected, which renders the transition irreversible. In the bifurcation diagrams one branch corresponds to the multipotent progenitor condition and the other to the B-lymphoid committed progenitor condition. Once EBF1 level accumulates beyond the threshold, an irreversible transition occurs from multipotent progenitor (LMPP cell, stable state I) to B-lymphoid LRP (pro-B cell, stable state II).

### Analysis of the core feedback loops

In order to gain a deeper insight into the regulatory mechanisms that generate the lineage commitment, in the following we explore the dynamics of the different subnetworks that compose the core feedback loops. The expression and activity of EBF1 must be finely regulated in order to maintain the commitment of B-lymphoid development and balancing B lymphopoiesis [31, 32]. Among the inhibitors of EBF1, ZNF521 has been suggested to play a crucial role suppressing *EBF1* activity [17, 18, 33]. More recent experiments have also shown that Ebf1 cooperates in a negative feedback loop to repress Zfp521 as differentiation proceeds [34]. Based on these experimental evidences, we examine the simplest feedback module, comprising only EBF1 and ZNF521, assuming heterodimeric bindings and positive autoregulations, S7 Fig (left panel). The associated mathematical model takes the form reported in Eq (2). Considering the nominal parameter values (S1 Table), the nullclines *d*ZNF521/*dt* = 0 and *d*EBF1/*dt* = 0 intersect at a single steady state (SS) corresponding to the LMPP point, S7 Fig (upper right panel). The bifurcation diagrams in S7 Fig report the steady-state values of EBF1 and ZNF521 as function of the environmental factor *T*_*EBF*1_. These diagrams show the existence of a single inaccessible turning point (LP), thus system Eq (2) cannot provide a bistable switch. On the basis of this analysis, other molecular interactions have to be included in the model in order to to obtain bistable dynamics.

The commitment of LMPPs to LRPs is essentially sustained by the three transcription factors E2A, EBF1 and PAX5 [35]. E2A acts upstream of EBF1 by modulating its expression and hence, in concert with EBF1, activates the transcription of the *PAX5* gene, leading to the reinforcement of the B-lymphoid commitment [36, 37]. Furthermore, EBF1 binds to and regulates in a direct manner the expression of PAX5 [38] which, in turn, stimulates the *EBF1* expression through a positive feedback loop by binding to the proximal *EBF1* promoter [39, 40]. Looking at the dynamical behavior of the EBF1/E2A/PAX5 positive feedback loop, see S8 Fig (left panel), it is possible to note that, although the cooperativity is increased by means of heterodimeric bindings between the TFs EBF1, E2A and PAX5, as described by Eq (3), the system cannot yield the classical S-shaped bifurcation plot that is characteristic of a bistable switch. Numerical continuation analysis, performed with respect to the environmental factor *T*_*EBF*1_, shows the existence of a single steady-state, suggesting that this feedback module fosters persistence in the committed state, S8 Fig (right panel).

*Flt3*-signaling cascade plays a very important role in B-cell development by priming early B-cell progenitors in order to proceed toward the B-cell differentiation, promoting the expression of IL-7 [41, 42]; indeed, mice lacking both FLT3- and IL-7R-derived signals fail to develop any B-cells [43]. Furthermore, the presence of a negative feedback on FLT3 transcription by PAX5 has been documented [44]. Therefore, another important issue is whether the negative feedback loop EBF1/PAX5/FLT3/IL-7R, illustrated in S9 Fig (left panel), may contribute to bistability. Bifurcation analysis performed with respect to *T*_*EBF*1_ values shows a single positive steady state for system Eq (4), relative to the fully committed state, in correspondence of which the expression of EBF1 and PAX5 are sustained at higher level and the FLT3 and IL-7R at lower ones, S9 Fig (right panel).

Expanding the model by assembling together the latter two feedback loops (see S10 Fig left panel) results in a reinforcement of the committed state, which does not provide the sought-after switch-like behavior, as derived by means of bifurcation analysis of model Eq (5), see S10 Fig (right panel). Analysis of the network obtained by combining the ZNF521-EBF1 loop with the EBF1/PAX5/FLT3/IL-7R one, see S11 Fig (left panel), does not appear to guarantee sufficient conditions for bistable behavior (the corresponding model is given in Eq (6): bifurcation analysis show the existence of positive steady-states relative to the committed lineage only, see S11 Fig (right panels). Also the combination of the feedback loop EBF1-ZNF521 with the EBF1/E2A/PAX5 one, see S12 Fig (left panel) and model Eq (7), does not yield bistability: bifurcation diagrams show that the system converges toward committed state.

Finally, we examine the dynamics generated by the hypothesized novel interaction, PAX5-ZNF521. Such interaction establishes a second feedback loop between EBF1 and ZNF521 mediated by PAX5, as illustrated in S13 Fig (left panel). This feedback structure, described by model Eq (8), is responsible for the irreversible commitment switch: bifurcation diagrams in S13 Fig (right panel) show typical curves exhibiting bistable irreversible behavior against variations in the transcriptional activation of EBF1.

### Model assessment with respect to experimental observations

**Fig 5:**
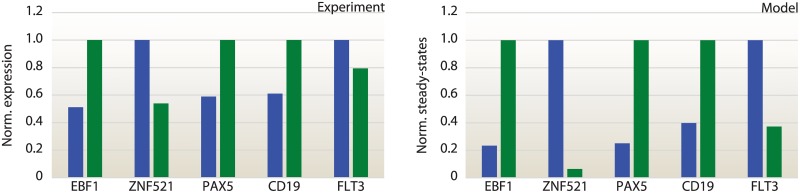
Comparison between experimental expression profiling and computed attractors. Each condition, for both experimental and model results, is normalized with respect to the given maximum value. It is possible to note a good agreement, in terms of bistable behavior, between the model prediction, the measured expression of the key factors involved in the commitment switch and the markers of the multipotent and the committed stage (FLT3 and CD19), respectively. Blue bars correspond to the LMPP stage, green bars to the LRP in the pro-B phase.

Similarly to other models of switch-like biological phenomena [11, 12], the proposed model is not aimed at providing quantitatively exact predictions of molecular concentrations, but rather to a system-level understanding of the mechanisms underlying the transitions between different operative conditions. Therefore, an assessment of the model plausibility can be done by means of a qualitative comparison of the *in silico* behavior with respect to the experimental bistable characteristics, rather than relying on some goodness-of-fit metrics. Thus, we compared the model steady-states with gene expression profiling datasets, focusing on the qualitative agreement between the model results and the gene profiling expressions (differentially gene expression results are reported in Fig 2 and S1 Fig). To confirm the phenomenological plausibility of the proposed model, we analyzed the results with respect to known molecular phenotypes, characterized by particular expression programs. We explicitly assessed the states of the attractors with gene expression profiles of differentiating multipotent progenitors toward B-lymphocytes. Human CD34^+^/lin^−^ and precursor B-cell subsets specific expression values were taken from [20, 21]. Fig 5 reports a comparison between experimental expression data and the model equilibrium points, highlighting only those factors whose expression varies by a significant amount across the different cellular states, namely, EBF1, PAX5 and ZNF521, and the markers FLT3 and CD19, expressed on the cell surface in the multipotent and committed stage, respectively.

For instance, in correspondence to the multipotent stage, the proposed model properly captures a higher expression of the LMPP phase promoters FLT3 and ZNF521; at the same time, the expression of the promoters of the lineage-restriction activity, EBF1, PAX5 and the surface B-precursor marker, CD19, is suppressed. In the B-lymphoid progenitors lineage, instead, the main fate B-cell development determinants EBF1, PAX5 and CD19 are strongly expressed in both experimental data and computational predictions, whereas FLT3 as ZNF521 are strongly repressed. These results suggest that the proposed commitment-switch model, derived by a combination of experimentally validated interactions and modeling hypotheses, is capable of reproducing the bistable behavior of the experimental system, in correspondence of the point in the parameter space defined by the solution proposed in S1 Table. This is not sufficient to determine the model plausibility: due to the significant inter-cellular variability of biomolecular circuits, a model can only be considered plausible if it is robust, i.e., the desired behavior is reproduced not only for a particular choice of the parameters, but over a non-trivial subset of the parameter-space [45–47].

Parametric sensitivity analysis showed that the bistable behavior of the proposed model is robust with respect to variations of the parameters involved in the dynamics of ZNF521 and EBF1. We have examined the range of parameter values in which bistable behavior is preserved, see S2 Table. In particular, the parameter *b*_4_, that expresses the strength of the inhibition of PAX5 on *ZNF521* in the nonlinear ODE model Eqs (1a)–(1j), can assume a wide range of values, log2 fold change value: [-0.524; 2.944], computed as the log2 ratio between the minimum and maximum admissible value, respectively, for which the system is bistable and the parameter used in the model, S1 Table. Furthermore, we have been able to identify domains of variation of the parameters involved in the dynamics of IL-7R and E2A, which are characterized by bistable dynamics. This is in agreement with the fact that these factors are known to play a crucial role in B lymphopoiesis, as reported in the S1 Text. The parametric robustness of the proposed model supports its plausibility, suggesting that the qualitative response does not depend on the particular choice of the parameters, but is rather a structural feature emerging from the topology of the interaction network and the kinetics of these interactions.

### Reprogramming strategies of B-lymphoid LRPs into multipotent progenitors

Directly increasing the *EBF1* transcriptional activity, *T*_*EBF*1_, the EBF1 levels are sufficient to induce PAX5 expression, which, in turn, decreases ZNF521 and FLT3 levels, see Fig 4. Consequently, *TCF3*, *EBF1* and *PAX5* are activated, leading to lymphoid-LRP commitment (specified by expression of the surface marker CD19). The dynamics of these master regulators yield two stable regimes, corresponding to the two lymphoid differentiation stages. In principle, by modulating the external factors that regulate the expression of *EBF1* and *ZNF521*, it is possible to revert the cell differentiation state from LRP to LMPP. To this aim, we need a strategy to make the system retrace back the classical S-shaped curves displayed in Fig 4. Since the bifurcations are driven by the master regulators EBF1, PAX5 and ZNF521, we have to act on these knobs to try to reprogram the cell fate.

**Fig 6:**
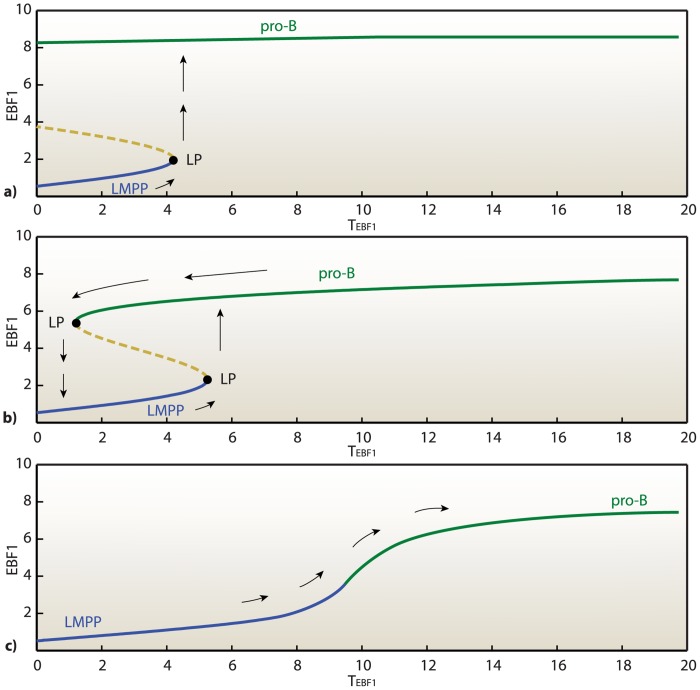
EBF1-dependence of the B-cell development bifurcation diagram w.r.t the ZNF521 transcriptional activation. **(a)** EBF1 versus external factor *T*_*EBF*1_ with no activation of ZNF521, *T*_*ZNF*521_ = 0. Overexpression of EBF1 is sufficient to switch the system from the LMPP state (low EBF1) to the pro-B (high EBF1) state, as indicated by the arrows. **(b)** EBF1 versus external factor *T*_*EBF*1_ with transcriptional activation of ZNF521, *T*_*ZNF*521_ = 0.12. A couple of limit points (LPs) defines a region of bistability for the proposed network. In this case, transcriptional activation of ZNF521 may convert the system from an irreversible to a reversible bistable switch. **(c)** EBF1 versus external factor *T*_*EBF*1_ with transcriptional activation of ZNF521, *T*_*ZNF*521_ = 0.3. Consistently, higher values of transcriptional activation of ZNF521 entailing a sharp delay in B-cell development, showing a more ultrasensitive response to higher values of *T*_*EBF*1_.

In some cases it is sufficient to modify the expression of a single transcription factor within a gene regulatory network to reprogram the cell fate [48]. An important question is whether the mutually inhibitory feedback from EBF1 to *ZNF521*, and the feedback from PAX5 to *FLT3* are essential to provide reinforcement of the commitment decision, and, more importantly, under which conditions the network can be modified to reverse the commitment. According to our model, the system can be reprogrammed from a state of lineage-restricted commitment by acting on the sole expression of ZNF521: this is evidenced by the bifurcation diagrams reported in Fig 6, showing that an increase in the expression of *ZNF521* transforms the irreversible switch into a reversible one. Under unperturbed conditions, the irreversibility of the switch is guaranteed by the repressing action of PAX5 on *ZNF521*, which is directly influenced by the expression level of *EBF1* but not by that of *ZNF521*. Therefore, to make the cell return to the preceding differentiation step, it is necessary to counteract this repression action by means of an exogenous transcriptional activation of *ZNF521*. As a consequence, the cell recovers the multipotent characteristics as self-renewal and differentiation into different lineages proceeding from a multipotent stage.

## Discussion

Unveiling the mechanisms underpinning multipotent cell lineage commitment is one of the main goals of systems biology. The heterogeneity of multipotent and progenitor cells produces sub-populations that express slightly different properties and propensities to commit towards the various lineages.

In hematopoiesis, cell fate mechanisms at the level of multipotent progenitors commitment toward B-lymphoid progenitors are still only partially elucidated and, to our knowledge, no model has so far been developed to describe the regulation of this differentiation stage. We have described these mechanisms by means of a phenomenological mathematical model, to quantitatively characterize the transcriptional dynamics regulating the commitment of multipotent lymphoid progenitors to lymphoid LRPs. The proposed model focuses on the role of the transcription co-factor ZNF521, a stem cell-associated protein that has been implicated in the regulation of the homeostasis of the immature compartment in a variety of normal and neoplastic tissues [17, 18, 34, 49–57]. In particular, previous work from our group suggested that ZNF521 can antagonize B-cell development and contribute to maintain the multipotency of primitive lympho-myeloid progenitors, by counteracting their EBF1-driven B-lymphoid commitment [18]. In the present study, the different stages of lineage commitment and differentiation have been described mathematically in terms of stable attractors in the state-space of the devised dynamical model.

It is important to remark that the main goal of our work is to propose a phenomenological model useful for the comprehension of the regulatory mechanisms underlying some steps of B-lymphoid cell differentiation; for this reason, the model is not intended as a tool for quantitative prediction of gene expression but, rather, for describing the qualitative response of the system during the various differentiation stages and for pinpointing the key molecular mechanisms that enable the lineage commitment. Due to the lack of experimental evidences regarding the nature of the molecular reactions, only heterodimeric pairwise interactions have been considered in the design of the model. Although the restriction to this class of models limits the generality of our approach, it has been deemed necessary to a) limit the arbitrariness that is inherent in the process of deriving a model from a network of interactions, and to b) focus our study on the discovery of novel putative molecular mechanisms responsible for the lineage commitment switch. An alternative approach would have been to investigate whether the bistable behavior might be explained by the introduction of high-order cooperative effects, for example in the feedback loop EBF1-ZNF512. However, it is well known that by choosing a suitable complex kinetics it easy to induce a bistable behavior, even in very simple reaction networks (even with only two species). Therefore, the results obtained through this modeling approach would not have been useful as a discriminating factor for guiding future biological experiments.

The main objective of this work is to identify novel putative transcriptional interactions that mediate an irreversible switch-like commitment of multipotent progenitors toward the B-lymphoid lineage. ZNF521 has been shown to interact with multiple molecular partners, that are likely to mediate its regulatory activities [17, 34, 49–52, 55]. Among these, the inhibition of EBF1 appears to be of particular relevance in the maintenance of the homeostasis of the early hematopoietic cell compartment by opposing the activity of *ZNF521*[18].

A first contribution of the work has consisted in the design of a mathematical model that recapitulates the known regulatory interactions between the molecular factors involved in the lineage commitment of B-lymphoid cells, including ZNF521. Subsequently, this *nominal* model has been exploited to evaluate the plausibility of newly hypothesized interactions, not yet experimentally validated. These putative interactions have been translated into different extended models, which have been analyzed to establish their capability to exhibit the sought-after switch-like behavior. Model assessment has also been performed by comparison between the simulated expression profiles and experimental gene expression datasets, showing a good qualitative agreement between the simulated and the experimentally observed behavior.

Model-based quantitative analysis has shown that the currently known experimentally validated interactions are not sufficient to explain the irreversible commitment dynamics; starting from this finding. Among the various hypotheses that have been investigated, the most conceivable resulted to be the existence of an inhibitory effect of PAX5 on the transcription of *ZNF521*. *PAX5* encodes a transcriptional regulator that has been shown to enable the B-cell-specific genetic program while repressing alternative ones [58]. Interestingly, Busslinger and colleagues have identified Pax5 binding within the *Zfp521* locus in pro B cells [59], although they have not evidenced regulation of *Zfp521* by Pax5. Direct repression of *Zfp521*, which is also highlighted in our model as the consequence of a possible direct interaction, has been reported in adipocyte progenitors by Kang et al. [34]. Considering that the expression of *EBF1* precedes that of *PAX5*, it can be hypothesized that the initial *ZNF521* repression by EBF1 is sufficient to lower the expression levels of the former, such that a further reduction of its expression in response to PAX5 may be undetectable by the gene profiling method used. Endowing our model with the PAX5 ⊣ ZNF521 interaction has resulted in the appearance of the commitment switch dynamics associated to the changes in the expression level of *EBF1*, *PAX5* and *ZNF521*.

Sensitivity analysis of the model parameters has allowed us to assess the robustness of the commitment switch with respect to inter-cellular parametric variability, thus supporting the plausibility of the proposed mathematical description of the regulatory interaction network. Moreover, sensitivity analysis has supported the identification of the key interactions driving the lineage commitment mechanism: the parameters involved in the dynamics of EBF1 and ZNF521, that are *a*_0_, *a*_2_, …, *a*_8_, *a*_10_, …, *a*_12_, *b*_0_, *b*_2_, …, *b*_4_, exhibit a substantial range of variation in which switch like behavior is preserved; a quantitative evaluation of such ranges of variation is illustrated in S3 Fig and in S2 Table. The parameters *f*_0_, …, *f*_3_, *j*_0_, *j*_1_, which are involved in the dynamics of IL-7R and E2A, can also determine a loss of the switch-like behavior if their values fall outside certain intervals; S4 Fig and S2 Table report the detailed analysis performed on this parameters and the bounds of the corresponding bistability intervals. Such analysis has suggested that these factors are critical for transitions between the two solutions. Instead, the remaining parameters, involved in the dynamics of GFI1, PU.1, IKAROS (only its basal expression has been considered) FLT3, CD19, PAX5 show unimodal behavior for single variation, i.e. each parameter value is characterized by a single CD19 homeostatic level starting point, S5 Fig.

Under nominal conditions, the commitment mechanism has resulted to be irreversible, as expected according to the experimental observations (see Fig 4). By exploiting the devised model, we have been able to design a possible reprogramming strategy from the LRP to LMPP stage, which may provide a valuable indication for future experimental investigations, (see Fig 6). The analyses conducted on our model suggest that a) increased transcription of *ZNF521* transforms the characteristic response of the commitment switch from an irreversible to a reversible form and b) the ZNF521-overxpressing cells may then be reverted to a multipotent state by repressing the expression of *EBF1*. It is interesting to notice, in this regard, that *Zfp521* is among the 36 HSC-associated genes that have been initially used by Riddell et al. [60] to reprogram committed mouse hematopoietic cells to induced HSCs. Although in subsequent experiments a more restricted cocktail of factors that did not include *Zfp521* has proven sufficient for this reprogramming, it will be of interest to test whether the combined overexpression of *ZNF521* and silencing of *EBF1* may indeed be effective in inducing the generation of multipotent progenitors from committed human B-lymphoid progenitors.

Dynamical models and the associated analysis methods provide effective tools for the comprehension of complex biological systems and for the *in silico* design of novel approaches to control their evolution. The regulatory mechanisms underlying multipotent cells differentiation represent a highly rewarding application field for such tools, as proven by previous analogous works, e.g. [12, 15, 16]. While the necessary steps of wet-lab experimental validation (such as the demonstration of the direct repression of *ZNF521* by PAX5 or the consequences of enforced expression of *ZNF521* in B-lymphoid progenitors that are currently underway) cannot be replaced by computational modeling and simulations, the presented results represent an advancement towards the comprehension of some key steps of B lymphopoiesis and will hopefully prove to be helpful in streamlining future experimental activity to better elucidate the hematopoietic regulatory mechanisms.

## Materials and Methods

### Determination of the transcriptional regulatory network

The first step in the generation of the proposed model consisted in the determination, based on the literature, of the key factors involved in the commitment of B-lymphoid lineage, and how these interact. The development of B lymphocytes from HSCs is organized as a multi-step programming process, turning on B-cell specific genes and silencing the expression of others. The commitment is initiated in correspondence of the LMPP stage, where the two transcription factors IKAROS and PU.1 start the early cellular specification [61, 62]. IKAROS and PU.1 can directly regulate the expression of FLT3, which is a marker of the LMPP stage [24, 25]. Our study also focused on the role of ZNF521 as an additional indicator of the multipotent developmental stage. At a later stage, the commitment of the lymphoid progenitor to B-lymphoid LRPs is essentially driven by three transcription factors: E2A, EBF1, and PAX5 [35]. This new stage is identified by a high expression level of CD19 on the cell surface. In the S1 Text, we provide a detailed analysis of the relevant literature, reporting the most up-to-date and significant experimental evidences concerning the LMPP to B-lymphoid LRP commitment mechanism. Furthermore, this biological information is arranged into a synthetic formal description, represented by the interaction network depicted in Fig 1.

### Mathematical models

#### Full Model

The gene regulatory network in Fig 1 has been translated into a mathematical model consisting of a set of nonlinear ordinary differential equations. The network dynamics have been modeled using simple expressions (e.g. quadratic and Michaelis-Menten-like mathematical terms), similarly to what is done with other phenomenological models of transcriptional networks, e.g. [12, 15]. Furthermore, for the sake of simplicity, we considered the following interpretation: the two cytokine signaling cascades on FLT3/Ras and IL-7R/Jak/Stat are described as direct interaction on the respective target. Hence, our model consists of only transcription factors and their interactions in a genetic control network, involving the ZNF521. We assume that the concentrations are in dimensionless units and the kinetic constants are in units of *s* ^−1^, and the Michaelis-Menten constants are dimensionless. The corresponding dynamical equations gives the following

$$\frac{dx_{\text{IKAROS}}}{dt}=i_{0}-\mu_{1}x_{\text{IKAROS}}$$ (1a)

$$\frac{dx_{\text{GFI1}}}{dt}=\frac{h_{0}+h_{1}x_{\text{IKAROS}}}{1+h_{1}x_{\text{IKAROS}}}-\mu_{2}x_{\text{GFI1}}$$ (1b)

$$\frac{dx_{\text{PU}.1}}{dt}=\frac{g_{0}+g_{1}x_{\text{PU}.1}}{1+g_{1}x_{\text{PU}.1}+g_{2}x_{\text{PU}.1}x_{\text{GFI1}}}-\mu_{3}x_{\text{PU}.1}$$ (1c)

$$\begin{array}{l} \frac{dx_{\text{FLT3}}}{dt}=\left(e_{0}+e_{1}x_{\text{PU}.1}+e_{2}x_{\text{IKAROS}}+e_{3}x_{\text{PU}.1}x_{\text{IKAROS}}\right)\\ \;\;\;\;\;\;\;\;\;\;\;\;\;\;\times 1/(1+e_{1}x_{\text{PU}.1}+e_{2}x_{\text{IKAROS}}+e_{3}x_{\text{PU}.1}x_{\text{IKAROS}}+e_{4}x_{\text{PAX5}}\\ \;\;\;\;\;\;\;\;\;\;\;\;\;\;+e_{5}x_{\text{PU}.1}x_{\text{PAX5}}+e_{6}x_{\text{IKAROS}}x_{\text{PAX5}})-\mu_{4}x_{\text{FLT3}} \end{array}$$ (1d)

$$\frac{dx_{\text{IL-7R}}}{dt}=\frac{f_{0}+f_{1}x_{\text{FLT3}}+f_{2}x_{\text{PU}.1}+f_{3}x_{\text{FLT3}}x_{\text{PU}.1}}{1+f_{1}x_{\text{FLT3}}+f_{2}x_{\text{PU}.1}+f_{3}x_{\text{FLT3}}x_{\text{PU}.1}}-\mu_{5}x_{\text{IL-7R}}$$ (1e)

$$\frac{dx_{\text{E2A}}}{dt}=\frac{j_{0}+j_{1}x_{\text{EBF1}}}{1+j_{1}x_{\text{EBF1}}}-\mu_{6}x_{\text{E2A}}$$ (1f)

$$\begin{array}{l} \frac{dx_{\text{ZNF521}}}{dt}=\frac{b_{0}+b_{1}T_{ZNF521}+b_{2}x_{\text{ZNF521}}}{1+b_{1}T_{ZNF521}+b_{2}x_{\text{ZNF521}}+b_{3}x_{\text{ZNF521}}x_{\text{EBF1}}+b_{4}x_{\text{EBF1}}x_{\text{PAX5}}}\\ \;\;\;\;\;\;\;\;\;\;\;\;\;\;\;\;\;\;-\mu_{7}x_{\text{ZNF521}} \end{array}$$ (1g)

$$\begin{array}{l} \frac{dx_{\text{EBF1}}}{dt}=(a_{0}+a_{1}T_{EBF1}+a_{2}x_{\text{EBF1}}+a_{3}x_{\text{PAX5}}+a_{5}x_{\text{IL-7R}}\\ \;\;\;\;\;\;\;\;\;\;\;\;\;\;\;+a_{6}x_{\text{EBF1}}x_{\text{IL-7R}}+a_{7}x_{\text{PU}.1}+a_{8}x_{\text{PAX5}}x_{\text{PU}.1}+a_{10}x_{\text{E2A}}\\ \;\;\;\;\;\;\;\;\;\;\;\;\;\;\;+a_{11}x_{\text{E2A}}x_{\text{EBF1}}+a_{12}x_{\text{E2A}}x_{\text{IL-7R}})\\ \;\;\;\;\;\;\;\;\;\;\;\;\;\;\;\times 1/(1+a_{1}T_{EBF1}+a_{2}x_{\text{EBF1}}+a_{3}x_{\text{PAX5}}+a_{4}x_{\text{ZNF521}}\\ \;\;\;\;\;\;\;\;\;\;\;\;\;\;\;+a_{5}x_{\text{IL-7R}}+a_{6}x_{\text{EBF1}}x_{\text{IL-7R}}+a_{7}x_{\text{PU}.1}+a_{8}x_{\text{PAX5}}x_{\text{PU}.1}\\ \;\;\;\;\;\;\;\;\;\;\;\;\;\;\;+a_{9}x_{\text{PU}.1}x_{\text{ZNF521}}+a_{10}x_{\text{E2A}}+a_{11}x_{\text{E2A}}x_{\text{EBF1}}\\ \;\;\;\;\;\;\;\;\;\;\;\;\;\;\;+a_{12}x_{\text{E2A}}x_{\text{IL-7R}})-\mu_{8}x_{\text{EBF1}} \end{array}$$ (1h)

$$\frac{dx_{\text{PAX5}}}{dt}=\frac{c_{0}+c_{1}x_{\text{EBF1}}+c_{2}x_{\text{E2A}}+c_{3}x_{\text{EBF1}}x_{\text{E2A}}}{1+c_{1}x_{\text{EBF1}}+c_{2}x_{\text{E2A}}+c_{3}x_{\text{EBF1}}x_{\text{E2A}}}-\mu_{9}x_{\text{PAX5}}$$ (1i)

$$\frac{dx_{\text{CD19}}}{dt}=\frac{d_{0}+d_{1}x_{\text{PAX5}}}{1+d_{1}x_{\text{PAX5}}}-\mu_{10}x_{\text{CD19}}$$ (1j)

where *x*_IKAROS_, *x*_GFI1_, *x*_PU.1_, *x*_FKT3_, *x*_IL-7R_, *x*_E2A_, *x*_ZNF521_, *x*_EBF1_, *x*_PAX5_, and *x*_CD19_ stand for the corresponding expression levels, the parameters *a*_0_, *b*_0_, *c*_0_, *d*_0_, *e*_0_, *f*_0_, *g*_0_, *h*_0_, *i*_0_ and *j*_0_ represent the basal production rate of each protein and the *μ*_*i*_ with *i* = 1, …, 10, define the degradation rates. Each protein is transcribed by RNA polymerase when it is bound either by one or more transcription factors. For each transcription factor involved in the model, we assume the level of protein approximately proportional to the mRNA levels. In Eqs (1h) and (1g) we assumed by *T*_*EBF*1_ and *T*_*ZNF*521_ the environmental factors activating the transcriptional activity of *EBF1* and *ZNF521*, respectively, in order to analyze the effects on early B lymphopoiesis lineage commitment, promoting in turn the activation of one of these key regulators.

#### EBF1/ZNF521 feedback

The dynamical equations corresponding to the simple feedback between EBF1 and ZNF521, reported in S7 Fig, assume the form:

$$\frac{dx_{\text{EBF1}}}{dt}=\frac{a_{0}+a_{1}T_{EBF1}+a_{2}x_{\text{EBF1}}}{1+a_{1}T_{EBF1}+a_{2}x_{\text{EBF1}}+a_{4}x_{\text{EBF1}}x_{\text{ZNF521}}}-\mu_{8}x_{\text{EBF1}}$$ (2a)

$$\frac{dx_{\text{ZNF521}}}{dt}=\frac{b_{0}+b_{1}T_{ZNF521}+b_{2}x_{\text{ZNF521}}}{1+b_{1}T_{ZNF521}+b_{2}x_{\text{ZNF521}}+b_{3}x_{\text{ZNF521}}x_{\text{EBF1}}}-\mu_{7}x_{\text{EBF1}}$$ (2b)

#### EBF1/E2A/PAX5 feedback

The dynamical equations corresponding to the EBF1/E2A/PAX5 positive feedback, reported in S8 Fig, are given by:

$$\frac{dx_{\text{EBF1}}}{dt}=\frac{a_{0}+a_{1}T_{EBF1}+a_{2}x_{\text{EBF1}}+a_{10}x_{\text{E2A}}+a_{11}x_{\text{E2A}}x_{\text{EBF1}}}{1+a_{1}T_{EBF1}+a_{2}x_{\text{EBF1}}+a_{10}x_{\text{E2A}}+a_{11}x_{\text{E2A}}x_{\text{EBF1}}}-\mu_{8}x_{\text{EBF1}}$$ (3a)

$$\frac{dx_{\text{E2A}}}{dt}=\frac{j_{0}+j_{1}x_{\text{EBF1}}}{1+j_{1}x_{\text{EBF1}}}-\mu_{6}x_{\text{E2A}}$$ (3b)

$$\frac{dx_{\text{PAX5}}}{dt}=\frac{c_{0}+c_{1}x_{\text{EBF1}}+c_{2}x_{\text{E2A}}+c_{3}x_{\text{EBF1}}x_{\text{E2A}}}{1+c_{1}x_{\text{EBF1}}+c_{2}x_{\text{E2A}}+c_{3}x_{\text{EBF1}}x_{\text{E2A}}}-\mu_{9}x_{\text{PAX5}}$$ (3c)

#### EBF1/PAX5/FLT3/IL-7R feedback

The dynamical equations corresponding to the feedback involving EBF1, PAX5, FLT3 and IL-7R, reported in S9 Fig, are expressed as follows:

$$\begin{array}{l} \frac{dx_{\text{EBF1}}}{dt}=\frac{a_{0}+a_{1}T_{EBF1}+a_{2}x_{\text{EBF1}}+a_{3}x_{\text{PAX5}}+a_{5}x_{\text{IL-7R}}+a_{6}x_{\text{EBF1}}x_{\text{IL-7R}}}{1+a_{1}T_{EBF1}+a_{2}x_{\text{EBF1}}+a_{3}x_{\text{PAX5}}+a_{5}x_{\text{IL-7R}}+a_{6}x_{\text{EBF1}}x_{\text{IL-7R}}}\\ \;\;\;\;\;\;\;\;\;\;\;\;\;\;\;-\mu_{8}x_{\text{EBF1}} \end{array}$$ (4a)

$$\frac{dx_{\text{PAX5}}}{dt}=\frac{c_{0}+c_{1}x_{\text{EBF1}}}{1+c_{1}x_{\text{EBF1}}}-\mu_{9}x_{\text{PAX5}}$$ (4b)

$$\frac{dx_{\text{FLT3}}}{dt}=\frac{e_{0}}{1+e_{4}x_{\text{PAX5}}}-\mu_{4}x_{\text{FLT3}}$$ (4c)

$$\frac{dx_{\text{IL-7R}}}{dt}=\frac{f_{0}+f_{1}x_{\text{FLT3}}}{1+f_{1}x_{\text{FLT3}}}-\mu_{5}x_{\text{IL-7R}}$$ (4d)

#### EBF1/E2A/PAX5/FLT3/IL-7R feedback

The dynamical equations corresponding to the feedback involving EBF1, E2A, PAX5, FLT3 and IL-7R, reported in S10 Fig, are given by:

$$\begin{array}{l} \frac{dx_{\text{EBF1}}}{dt}=(a_{0}+a_{1}T_{EBF1}+a_{2}x_{\text{EBF1}}+a_{3}x_{\text{PAX5}}+a_{5}x_{\text{IL-7R}}\\ \;\;\;\;\;\;\;\;\;\;\;\;\;\;\;+a_{6}x_{\text{EBF1}}x_{\text{IL-7R}}+a_{10}x_{\text{E2A}}+a_{11}x_{\text{E2A}}x_{\text{EBF1}}+a_{12}x_{\text{E2A}}x_{\text{IL-7R}})\\ \;\;\;\;\;\;\;\;\;\;\;\;\;\;\;\times 1/(1+a_{1}T_{EBF1}+a_{2}x_{\text{EBF1}}+a_{3}x_{\text{PAX5}}+a_{5}x_{\text{IL-7R}}\\ \;\;\;\;\;\;\;\;\;\;\;\;\;\;\;+a_{6}x_{\text{EBF1}}x_{\text{IL-7R}}+a_{10}x_{\text{E2A}}+a_{11}x_{\text{E2A}}x_{\text{EBF1}}+a_{12}x_{\text{E2A}}x_{\text{IL-7R}})\\ \;\;\;\;\;\;\;\;\;\;\;\;\;\;\;-\mu_{8}x_{\text{EBF1}} \end{array}$$ (5a)

$$\frac{dx_{\text{E2A}}}{dt}=\frac{j_{0}+j_{1}x_{\text{EBF1}}}{1+j_{1}x_{\text{EBF1}}}-\mu_{6}x_{\text{E2A}}$$ (5b)

$$\frac{dx_{\text{PAX5}}}{dt}=\frac{c_{0}+c_{1}x_{\text{EBF1}}+c_{2}x_{\text{E2A}}+c_{3}x_{\text{EBF1}}x_{\text{E2A}}}{1+c_{1}x_{\text{EBF1}}+c_{2}x_{\text{E2A}}+c_{3}x_{\text{EBF1}}x_{\text{E2A}}}-\mu_{9}x_{\text{PAX5}}$$ (5c)

$$\frac{dx_{\text{FLT3}}}{dt}=\frac{e_{0}}{1+e_{4}x_{\text{PAX5}}}-\mu_{4}x_{\text{FLT3}}$$ (5d)

$$\frac{dx_{\text{IL-7R}}}{dt}=\frac{f_{0}+f_{1}x_{\text{FLT3}}}{1+f_{1}x_{\text{FLT3}}}-\mu_{5}x_{\text{IL-7R}}$$ (5e)

#### ZNF521/EBF1/PAX5/FLT3/IL-7R feedback

The dynamical equations corresponding to the module constituted by ZNF521/EBF1/PAX5/FLT3/IL-7R, reported in S11 Fig, assume the form:

$$\begin{array}{l} \frac{dx_{\text{EBF1}}}{dt}=(a_{0}+a_{1}T_{EBF1}+a_{2}x_{\text{EBF1}}+a_{3}x_{\text{PAX5}}\\ \;\;\;\;\;\;\;\;\;\;\;\;\;\;\;+a_{5}x_{\text{IL-7R}}+a_{6}x_{\text{EBF1}}x_{\text{IL-7R}})\\ \;\;\;\;\;\;\;\;\;\;\;\;\;\;\;\times (1+a_{1}T_{EBF1}+a_{2}x_{\text{EBF1}}+a_{3}x_{\text{PAX5}}+a_{4}x_{\text{EBF1}}x_{\text{ZNF521}}\\ \;\;\;\;\;\;\;\;\;\;\;\;\;\;\;+a_{5}x_{\text{IL-7R}}+a_{6}x_{\text{EBF1}}x_{\text{IL-7R}})-\mu_{8}x_{\text{EBF1}} \end{array}$$ (6a)

$$\frac{dx_{\text{ZNF521}}}{dt}=\frac{b_{0}+b_{1}T_{ZNF521}+b_{2}x_{\text{ZNF521}}}{1+b_{1}T_{ZNF521}+b_{2}x_{\text{ZNF521}}+b_{3}x_{\text{EBF1}}}-\mu_{7}x_{\text{ZNF521}}$$ (6b)

$$\frac{dx_{\text{PAX5}}}{dt}=\frac{c_{0}+c_{1}x_{\text{EBF1}}}{1+c_{1}x_{\text{EBF1}}}-\mu_{9}x_{\text{PAX5}}$$ (6c)

$$\frac{dx_{\text{FLT3}}}{dt}=\frac{e_{0}}{1+e_{4}x_{\text{PAX5}}}-\mu_{4}x_{\text{FLT3}}$$ (6d)

$$\frac{dx_{\text{IL-7R}}}{dt}=\frac{f_{0}+f_{1}x_{\text{FLT3}}}{1+f_{1}x_{\text{FLT3}}}-\mu_{5}x_{\text{IL-7R}}$$ (6e)

#### ZNF521/EBF1/E2A/PAX5 feedback

The dynamical equations corresponding to the module constituted by ZNF521/EBF1/E2A/PAX5, reported in S12 Fig, assume the form:

$$\begin{array}{l} \frac{dx_{\text{EBF1}}}{dt}=(a_{0}+a_{1}T_{EBF1}+a_{2}x_{\text{EBF1}}+a_{3}x_{\text{PAX5}}\\ \;\;\;\;\;\;\;\;\;\;\;\;\;\;\;+a_{1}0x_{\text{E2A}}+a_{1}1x_{\text{EBF1}}x_{\text{E2A}})\\ \;\;\;\;\;\;\;\;\;\;\;\;\;\;\;\times (1+a_{1}T_{EBF1}+a_{2}x_{\text{EBF1}}+a_{3}x_{\text{PAX5}}+a_{4}x_{\text{EBF1}}x_{\text{ZNF521}}\\ \;\;\;\;\;\;\;\;\;\;\;\;\;\;\;+a_{1}0x_{\text{E2A}}+a_{1}1x_{\text{EBF1}}x_{\text{E2A}})-\mu_{8}x_{\text{EBF1}} \end{array}$$ (7a)

$$\frac{dx_{\text{ZNF521}}}{dt}=\frac{b_{0}+b_{1}T_{ZNF521}+b_{2}x_{\text{ZNF521}}}{1+b_{1}T_{ZNF521}+b_{2}x_{\text{ZNF521}}+b_{3}x_{\text{EBF1}}}-\mu_{7}x_{\text{ZNF521}}$$ (7b)

$$\frac{dx_{\text{E2A}}}{dt}=\frac{j_{0}+j_{1}x_{\text{EBF1}}}{1+j_{1}x_{\text{EBF1}}}-\mu_{6}x_{\text{E2A}}$$ (7c)

$$\frac{dx_{\text{PAX5}}}{dt}=\frac{c_{0}+c_{1}x_{\text{EBF1}}+c_{2}x_{\text{E2A}}+c_{3}x_{\text{EBF1}}x_{\text{E2A}}}{1+c_{1}x_{\text{EBF1}}+c_{2}x_{\text{E2A}}+c_{3}x_{\text{EBF1}}x_{\text{E2A}}}-\mu_{9}x_{\text{PAX5}}$$ (7d)

#### EBF1/PAX5/ZNF521 feedback

The dynamical equations corresponding to the EBF1/PAX5/ZNF521 feedback, reported in S13 Fig, assume the form:

$$\begin{array}{l} \frac{dx_{\text{EBF1}}}{dt}=\frac{a_{0}+a_{1}T_{EBF1}+a_{2}x_{\text{EBF1}}+a_{3}x_{\text{PAX5}}}{1+a_{1}T_{EBF1}+a_{2}x_{\text{EBF1}}+a_{3}x_{\text{PAX5}}+a_{4}x_{\text{EBF1}}x_{\text{ZNF521}}}\\ \;\;\;\;\;\;\;\;\;\;\;\;\;\;\;-\mu_{8}x_{\text{EBF1}} \end{array}$$ (8a)

$$\begin{array}{l} \frac{dx_{\text{ZNF521}}}{dt}=\frac{b_{0}+b_{1}T_{ZNF521}+b_{2}x_{\text{ZNF521}}}{1+b_{1}T_{ZNF521}+b_{2}x_{\text{ZNF521}}+b_{3}x_{\text{EBF1}}+b_{4}x_{\text{EBF1}}x_{\text{PAX5}}}\\ -\mu_{7}x_{\text{ZNF521}} \end{array}$$ (8b)

$$\frac{dx_{\text{PAX5}}}{dt}=\frac{c_{0}+c_{1}x_{\text{EBF1}}}{1+c_{1}x_{\text{EBF1}}}-\mu_{9}x_{\text{PAX5}}$$ (8c)

Numerical simulations and bifurcation analysis have been performed by using Matlab and the Matcont continuation package [63], respectively. In order to get a biologically plausible model, we tuned the values of the model parameters such that the simulated response of the model matched the qualitative behavior of the experimental system, according to the experimental data reported in a set of microarray gene experiments. Given a certain network topology, along with a tentative model of its dynamics and admissible intervals of its parameters, the Bifurcation Discovery Tool (BDT) allows the computation of parameter set values that give rise to bifurcations [64]. The parameter space is explored by means of a genetic algorithm, seeking Hopf bifurcations, turning points and bistable switches. In particular, starting from an ODE-based reaction network, BDT allows an user to choose the parameters to be searched, the admissible parameter ranges, and the nature of the bifurcation to be sought. As a result, the tool will return the parameter values for which the sought-after behavior is observed. We have identified a set of parameter values that correspond to a bistable behavior of the network and to a system response that is in agreement with experimental measurements.

### Microarray expression profiling

We considered gene expression given as supplementary data in [20, 21]. In the latter works, the authors treated the development of precursor B-cells from hemopoietic stem cells toward differentiation through a number of stages in the bone marrow before their migration to the periphery as naive mature B lymphocytes. (ArrayExpress Database, accession no. E-MEXP-384). B-cells progenitors were obtained from bone marrow samples of healthy children (age 3–16). Lineage-depleted (lin^−^) human cells enriched for expressing CD34 (CD34^+^ cells) were obtained from umbilical cord blood, since it is impossible to obtain enough CD34^+/^lin^−^ cells from bone marrow samples. In general, lin^−^ cells identify all the stem and progenitor cells for which mature cell lineage markers expression is undetectable. We have performed gene expression profiling using Affymetrix Expression Console, applying a robust multi-array normalization, and Transcriptome Analysis Console for differential expression analysis. Expression Console and TAC Softwares can be freely downloaded from the Affymetrix website.

## Supporting Information

S1 Text Supporting Text. (PDF)

S1 Fig Volcano plot showing gene level differential expression between the two conditions CD34^+^/lin^−^ and pro-B vs. significance value. All the points having a fold change (linear) greater than 2 and p-value < 0,05 indicate points-of-interest characterized by both large-magnitude fold-changes as well as high statistical significance (-log10 of p-value). All that points having a fold change (linear) less than 2 and p-value greater than 0,05 are shown in gray. Red: up in CD34^+^/lin^−^ vs pro-B; green: down in CD34^+^/lin^−^ vs pro-B. Highlighted are shown the factors involved in the proposed model confirming a well agreement between experimental and computed results.
(EPS)

S2 Fig Time profile concentrations of FLT3, ZNF521, EBF1, PAX5 and CD19 for the two cellular stages, indicating the final steady state values. **(a)** When the ZNF521 is activated, the LMPPs specified genes are highly expressed arresting the development of B cells. Differently, **(b)** when no factor stimulates the expression of ZNF521, the system is driven towards the lymphoid lineage fate, resulting in a higher expression of the B-lymphoid LRP specified genes.
(EPS)

S3 Fig CD19 bifurcation analysis with respect to the parameters involved in EBF1 and ZNF521 transcriptional dynamics. The parameters associated to EBF1 transcription, *a*_0_, *a*_2_, …, *a*_8_, *a*_10_, …, *a*_12_, and all the parameters involved in ZNF521 transcription, *b*_0_, *b*_2_, …, *b*_4_ exhibit an interval where bistable solutions can be found.
(EPS)

S4 Fig CD19 bifurcation analysis with respect to the parameters involved in IL-7R, PU.1, IKAROS and E2A transcriptional dynamics. The parameters associated to IL-7R transcription, *f*_0_, …, *f*_3_ and to E2A transcription, *j*_0_, *j*_1_ exhibit an interval where bistable solutions can be found. Individual variations of the kinetic parameters describing the PU.1 dynamics, *g*_0_, …, *g*_2_, the GFI1 dynamics, *h*_0_, *h*_1_ and the basal expression of IKAROS, *i*_0_, instead, do not give rise to bistability.
(EPS)

S5 Fig CD19 bifurcation analysis with respect to the parameters involved in PAX5, CD19 and FLT3 transcriptional dynamics. The parameters associated to PAX5 transcription, *c*_0_, …, *c*_3_, to CD19 transcription *d*_0_, *d*_1_ and all the parameters involved in FLT3 transcription, *e*_1_, …, *e*_6_ (with the only exception of its basal expression, *e*_0_), do not give rise to bistability.
(EPS)

S6 Fig Comparison of the proposed model with different hypothetical configurations. Bifurcation study proves the crucial role of the inhibition of PAX5 on ZNF521 expression to admit the existence of commiment switching between LMPP and pro-B stages in B-lymphocyte development. Starting numerical continuation from the LMPP stage, let us impose the conditions related to the case of reversible commitment switch, for *T*_*ZNF*521_ = 0.12. **Model A.** As already seen, ZNF521 inhibition through PAX5 generates a bistable commitment switch between LMPPs (lower values of CD19 expression) and LRP cells (greater values of CD19 expression). **Model B.** Removing ZNF521 inhibition through PAX5, the effects of inhibition of ZNF521 through the factor E2A have been considered. In this case, the system is characterized by a single CD19 expression level, preventing the occurrence of switching behavior. **Model C.** Removing ZNF521 inhibition through PAX5, the effects of ZNF521 repression through IKAROS have been considered. Also in this case, CD19 reaches a single expression level.
(EPS)

S7 Fig Dynamical analysis of the EBF1/ZNF521 feedback module. The EBF1-ZNF521 mutual inhibition, with autoregulatory interactions; the environmental factors, which promote the transcriptional activity of EBF1 and ZNF521 are indicated as *T*_*EBF*1_ and *T*_*ZNF*521_, respectively. The intersection among the nullclines *d*ZNF521/*dt* = 0 and *d*EBF1/*dt* = 0, with parameters given by S1 Table, identifies a single stable point (SS) corresponding to the LMPP point (upper right panel). Bifurcation analysis performed promoting the transcriptional activity of EBF1 shows the existence of a single inaccessible turning point (LP), for negative values of *T*_*EBF*1_ (lower right panels). Solid line denotes stable steady-states, dashed line denotes unstable ones.
(EPS)

S8 Fig Dynamical analysis of the EBF1/E2A/PAX5 feedback module. The positive feedback loop constituted by EBF1, E2A and PAX5, with autoregulatory loop and the environmental factor, *T*_*EBF*1_, acting on EBF1 transcription. Bifurcation analysis shows the expression levels of the corresponding factor as functions of *T*_*EBF*1_. It is possible to note that this feedback sustains the committed state, admitting only one stable positive point (right panels).
(EPS)

S9 Fig Dynamical analysis of the EBF1/PAX5/FLT3/IL-7R feedback module. As suggested by experimental findings, EBF1, PAX5, FLT3 and IL-7R form a negative loop (left panel). Bifurcation analysis performed with respect to *T*_*EBF*1_ values shows that this feedback admits a single positive steady state, relative to the committed state, in correspondence of which the expression of EBF1 and PAX5 are sustained at higher level and the FLT3 and IL-7R at lower ones (right panels). Solid line denotes stable steady-states, dashed line denotes unstable ones.
(EPS)

S10 Fig Dynamical analysis of the EBF1/E2A/PAX5/FLT3/IL-7R feedback module. The feedback loop comprising the positive loop involving EBF1, E2A and PAX5 and the negative one constituted by EBF1, PAX5, FLT3 and IL-7R (left panel). Bifurcation diagrams show the expression levels of the involved factors as functions of *T*_*EBF*1_. It is possible to observe a reinforcement of the committed state, which does not provide the sought-after switch-like behavior (right panels).
(EPS)

S11 Fig Dynamical analysis of the ZNF521/EBF1/PAX5/FLT3/IL-7R feedback module. The module constituted by the mutual inhibition between ZNF521 and EBF1, with autoregulatory loops, and the negative feedback formed by EBF1/PAX5/FLT3/IL-7R (left panel), does not appear to be sufficient to bistability behavior. Bifurcation analysis performed versus *T*_*EBF*1_ show the existence of only positive steady-states relative to the committed lineage (right panels). Solid line denotes stable steady-states, dashed line denotes unstable ones.
(EPS)

S12 Fig Dynamical analysis of the ZNF521/EBF1/PAX5/E2A feedback module. The module constituted by the mutual inhibition between ZNF521 and EBF1, with autoregulatory loops, and the positive feedback loop that sustain the committed state, EBF1/E2A/PAX5 (left panel), are unable to capture the required switch-like behavior. Observing the bifurcation diagrams (right panels) for consistent values of *T*_*EBF*1_ the system converges to the committed state. Solid line denotes stable steady-states, dashed line denotes unstable ones.
(EPS)

S13 Fig Dynamical analysis of the EBF1/PAX5/ZNF521 feedback module. The EBF1/ZNF521 mutual inhibition, with autoregulatory loops, is enriched by a further interaction through the key factor PAX5. Observing the bifurcation diagrams (right panels) with respect to the *T*_*EBF*1_ values, this feedback induces irreversible bistable behavior. Solid line denotes stable steady-states, dashed line denotes unstable ones.
(EPS)

S1 Table Parameter values used in the dynamical equations. Set of parameter values in correspondence of which the main model exhibits a classic bistable behavior.
(PDF)

S2 Table Parameter sensitivity coefficients for bistability. The parameter sensitivity for bistability is defined as the log2 fold change of the ratio between the max (min) parameter value for which the system is bistable and the parameter used in the model, S1 Table.
(PDF)

S3 Table Parameter values used in the model B and C bifurcation studies. Parameters values used as different configurations of the original transcriptional regulatory network, considering reasonable regulatory gene hypotheses.
(PDF)
