# Supplementary material for: Computational Modeling of a Transcriptional Switch Underlying B-Lymphocyte Lineage Commitment of Hematopoietic Multipotent Cells
Source: PLoS One. 2015 Jul 13;10(7):e0132208. doi: 10.1371/journal.pone.0132208 (PMC4500571; doi:10.1371/journal.pone.0132208)
Supplement: S1 Text — (PDF) [file pone.0132208.s001.pdf]

Supporting Text

Luca Salerno[1,◑], Carlo Cosentino[1,◑,*], Giovanni Morrone[2], Francesco Amato[1]

1 Laboratory of Biomechatronics, Department of Experimental and Clinical Medicine, University Magna Græcia of Catanzaro, Catanzaro, Italy and 2 Laboratory of Molecular Haematopoiesis and Stem Cell Biology, Department of Experimental and Clinical Medicine, University Magna Græcia of Catanzaro, Catanzaro, Italy

◑These authors contributed equally to this work.
*** carlo.cosentino@unicz.it**

Basic modelling methodology

Building model for a biochemical network requires several different steps or tasks to be engaged. The theoretical modelling has to be combined with biological experiments for an effective and useful approach. Important steps in building bio-mathematical models are:

(i) **Define the involved players and interactions.** Firstly, determining which factors are involved in different biological processes, and how these interact.

(ii) **Describe in a mathematical formalism the network involving species and interactions.** A quantitative model of a biochemical network is represented as a dynamical system where molecular concentrations are the state variables and their interactions are described explicitly by mathematical functions.

(iii) **Parameter estimation.** Since each point in parameter space, given by a set of parameter values, defines the dynamics on the state space, it is fundamental to estimate the parameter values that are relevant for a specific biological network.

(iv) **Analyze the dynamical behavior.** Models provide an ideal benchmark to test the effect of concentration and operating parameters, to study the effect of network perturbation where molecules or interactions are removed from the system, which then can be compared with knock-out experiments, or provide biological predictions from the model.

Commitment switch of LMPPs toward B lymphoid lineage: involved players and interactions

In correspondence of the LMPP stage, the two transcription factors, IKAROS and PU.1 are believed to be responsible of the early lymphopoietic specification. The *IKAROS* gene encodes a family of zinc finger transcription factors that are essential for the generation of fetal B and T cell progenitors. Germline mutation of the *IKAROS* DNA-binding domain in homozygous mice showed the influence of IKAROS zinc finger transcription factor (IKZF1) for hematopoietic stem cell function and renewal, and promotion of the differentiation of T and B lymphocytes and natural killer cells [1–3]. In other studies, IKAROS was shown to be a crucial regulator in the commitment of

LMPPs into the lymphoid lineage playing a key role in the early lymphocyte commitment [4]. In particular, LMPPs derived from IKAROS knockout mice, T-cells but not B-cells, are generated late in mouse development, and the latter do not express *FLT3*, *IL-7R*, *RAG1* and *RAG2*, important genes for the lymphoid commitment [5]. Beside these functions, IKAROS can either be involved at later stages for V(D)J recombination[1], depending on the cooperation with different coactivators or corepressors [7]. For example, in T cells, IKAROS has been shown to yield epigenetic modifications causing chromatin remodelling that result in either repression or activation of sets of targets genes [8]. However, how IKAROS mediates the regulation of its target genes during the fate commitment of LMPPs is not yet fully understood.

The transcription factor PU.1 plays also a key role for the commitment of LMPPs towards the lymphoid lineage. This protein, that is encoded in humans by the SPI1 gene and belongs to the Ets family of transcription factors, is uniquely required for the generation of B-cells and macrophages. PU.1 regulates the transcription of the M-CSF receptor (c-fms) gene and consequently modulates the responsiveness of monocyte progenitors to M-CSF [9, 10]. The direct interaction between PU.1 and GATA-1 prevents the induction, by the latter factor, of an erythroid cell fate in uncommitted progenitors [11, 12]. Applying different levels of PU.1 in a common progenitor population, it has been experimentally shown that lower levels of PU.1 favor B-lymphoid rather than macrophage development [13]. Recently [14], it has been also shown that PU.1 can directly regulate the expression of *FLT3* and, consequently, is involved in the development of LMPPs. Moreover, PU.1 has been shown to positively autoregulate its own expression driving cell fate to a myeloid fate at the expense of the lymphoid one [15], and it has also been implicated in the regulation of IL-7R transcription in B-cell progenitors [16]. Another relevant transcription factor in the control of B-cell development is GFI1, a target of IKAROS, that has been shown to repress the transcription of the *SPI1* gene thereby maintaining the levels of PU.1 low, and compatible with the B-lymphoid rather than myeloid differentiation [17]. Such IKAROS-GFI1-SPI1 feedback must be properly balanced, since PU.1 must however still be able to participate in the activation of IL-7R that is required for the progression of the development from LMPPs to B lymphoid lineage [18, 19].

The next early regulatory event contributing to B-cell commitment is expression of the receptor tyrosine kinase FLT3. *FLT3* also known as *CD135*, is a type III receptor tyrosine kinase. *FLT3* is a proto-oncogene, and a variety of mutations that results in the productions of constitutively active forms of the receptor can lead to the development of aggressive myeloid and lymphoid leukemias. When this receptor binds to Flt3L it forms a homodimer that activates its intrinsic tyrosine kinase activity, which in turn phosphorylates and activates signal transduction molecules that propagate the signal in the cell. In the mouse, MPPs and CLPs express high surface levels of this receptor. Flt3 expression is therefore used as a marker to differentiate HSCs, which are $Flt3^{-}$, from MPPs, which are $FLT3^{+}$ [20]. $Flt3^{-/-}$ mice show a severe deficiency in B-cell progenitors, and the mutant HSCs have lost their ability to reconstitute lymphoid and myeloid cells in recipient mice [21]. FLT3 signaling via RAS-dependent pathway plays an important role in B-cell development. Using mice expressing a dominant negative form of RAS, it has been demonstrated that RAS-mediated signaling plays a critical role in the development of lymphoid lymphoid-restricted progenitors (LRPs) [22]. This developmental block is correlated with that found in $FLT3^{-/-}$ mice, suggesting that *Flt3* is a crucial upstream activator of Ras in early B-cell progenitors. The Ras-dependent pathway inhibits the expression of the modulators of cytokine signaling,

[1] V(D)J recombination is a mechanism of genetic recombination in the early stages of immunoglobulin (Ig) and T cell receptors (TCR) production of the immune system. V(D)J recombination takes place in the primary lymphoid tissue, [6].

SOCS2 and SOCS3, thereby promoting STAT5-dependent pro B-cell differentiation through the enhanced expression of IL-7R [22]. This suggests a network configuration in which FLT3/Ras-dependent signaling cascade plays a very important role in B-cell development by priming early B-cell progenitors for subsequent STAT5-dependent B-cell differentiation.

IL-7 receptor is a heterodimer made up of two different smaller protein chains, IL-7Rα (CD127) and the common-γ chain receptor (CD132) [23]. The development of B-lymphoid LRPs in the bone marrow requires the signaling through the α-chain of this receptor (IL-7Rα) [24, 25]. Mice lacking both FLT3- and IL-7R-derived signals fail to develop any B-cells; these results suggest the strong possibility that these two cytokine signaling may activate the expression or modulate the activity of downstream key transcriptional regulators [26]. IL-7Rα-deficient mice show a more evident impairment of B-cell development than IL-7R$\gamma^{-/-}$ mice [27].

The differentiation of lymphoid progenitors to committed early B lymphopoiesis is essentially guaranteed by the three transcription factors E2A, EBF1, and PAX5, [28]. The *TCF3* gene encodes by differential splicing two transcription factors E47 and E12 (commonly referred to as E2A), that act both upstream of EBF1 by regulating its expression and also, in concert with EBF1, activate the transcription of the *PAX5* gene, leading to the further progression in the B-lymphoid development [29, 30]. In contrast to PU.1 and IKAROS deficient MPPs, which cannot differentiate further, common lymphoid progenitors (CLPs) are present in the absence of E2A, but are strongly reduced in numbers and show reduced surface expression of IL-7R [31, 32]. E-box [2] sequences, i.e. E2A-binding sites, are present in the *EBF1* promoter, revealing that *EBF1* is an E2A target gene, [34].

Similar to E2A, EBF1 is also known to play a crucial role in initiating B-cell development; this factor may be considered a master regulator of B-cell development. Inactivation of *EBF1* results in a similar arrest, as observed in E2A mutant mice, at the stage of a lymphoid progenitor that express early B-lymphoid markers such as FLT3, IL-7R and B220 [35], but fail to express B-cell genes such as mb-1, B29, λ5 and VpreB1 [36, 37]. Enforced expression of Ebf1 in mice deficient of E2a, Il-7, Il-7Rα, Ikaros or Pu.1 is sufficient to overcome the developmental block in these mice suggesting that Ebf1 acts downstream of these effectors [35, 38–40]. Recent reports have also highlighted the EBF1-mediated inhibition of B lineage-inappropriate genes, which contributes to restrict the lineage choices of lymphoid progenitors to the B-cell lineage independently of the B-cell commitment factor PAX5 [41]. The expression of EBF1 is regulated by two different promoters. The activity of the distal promoter EBF1-α is regulated by Il-7 signaling, E2A and by EBF1 autoregulation, whereas the proximal promoter EBF1-β is controlled by PAX5, ETS1 and PU.1 [42]. Furthermore, expression and binding site analyses suggest that EBF1 binds to and regulates directly the expression of transcription factor PAX5, [43, 44], which arises to maximal levels in pro-B-cells [45].

The *PAX5* gene encoding the transcription factor PAX5 appears to function downstream to PU.1, IKAROS, E2A, and EBF1, playing a crucial role in the final steps of B-cell development [46]. PAX5 plays a dual role by repressing B lineage-specific genes while, simultaneously, repressing B lineage-inappropriate genes. In PAX5 mutant bone marrow, B220^{+} B-cell progenitors expressing normal levels of PU.1, IKAROS, E2A and EBF1 transcripts as well as of RAG1, RAG2, and the surrogate light chain genes are generated but are unable to differentiate *in vitro* in the presence of stromal cells and IL-7. These cells do not express CD19 [47], and although unable to generate mature B-cells, display the ability to differentiate into several hematopoietic cell types either *in*

[2] E-box. Enhancer box is a DNA sequence found upstream of some promoter regions in eukaryotes that acts as a protein-binding site. Once the transcription factors bind to the promoters through the E-box, other enzymes can bind to the promoter and facilitate transcription from DNA to mRNA, [33].

vitro, in the presence of appropriate cytokines, or *in vivo* after transplantation into recipient mice. PAX5 stimulates *EBF1* expression through a positive feedback loop by binding to the proximal *EBF1* promoter [42, 45], which further enhances the B-cell commitment process of B-lymphoid progenitors [48]. Intriguingly, a negative feedback on FLT3 transcription by PAX5 has been documented: PAX5-deficient B-cell progenitors express abundant FLT3 that is strongly silenced upon the reintroduction of PAX5, whereas enforced expression of FLT3 in wild-type progenitors significantly impairs B-cell development. This is crucial for B-cell lineage commitment, since would seem directly repress genes associated with multipotency [49]. PAX5 has also been involved in human B-cell malignancies, as it is mutated, involved in translocation that generate aberrant fusion genes, or deregulated by epigenetic modifications in a subset of acute lymphoblastic leukemias and non-Hodgkin lymphomas [50].

The B-lymphocyte antigen CD19, the main PAX5 gene target, is a protein expressed on the surface of all B-lymphoid cells with the exception of terminally differentiated plasma cells and has been implicated as a signal-transducing receptor in the control of proliferation and differentiation. Lymphoid LRPs are the first B-cells that express Igα and Igβ on the cell surface and at this stage they start to express CD19, marking the earliest progenitors committed to B lineage [51].

The cell fate commitment from LMPPs towards the lymphoid lineage development of B lymphocytes is thus a tightly regulated process that requires the coordinated regulation of transcriptional reprogramming factors. As illustrated above, this is guaranteed by the concerted action of a transcriptional network and epigenetic modifications within which the factor EBF1, represents one of the main players. During the commitment of B-lymphoid development, the expression and activity of EBF1 must be modulated in order to maintain the pool of progenitors and establish a proper balance between B and T lymphopoiesis [52]. Among the inhibitors of EBF1 [53, 54], the zinc finger protein 521 (ZNF521 humans, Zfp521 in mice, also known as Evi3 or EHZF) is suggested to represent a key factor. ZNF521 is a protein with 30 zinc finger motifs with characteristics of transcriptional co-regulator first detected in a B-cell lymphoma mouse model [55] and then identified as one of the major transcripts of human primitive hematopoietic progenitors [56]. Recent studies have also shown that, in addition to hematopoietic stem and early progenitor cells, ZNF521 is expressed also in neural stem and progenitor cells, mesenchymal stem cells, osteoblasts progenitors and chondrocytes [57–59]. In all these types of cells, ZNF521 appears to control the differentiation of immature cells by modulating the activity of specific transcription factors. ZNF521 suppresses *EBF1* activity through the C-terminal zinc fingers, and modulates the development of B cells [53]. In particular, it appears that ZNF521 can bind to *EBF1* promoter through its C-terminal domain, and this interaction is required for suppression of EBF1. The silencing of *ZNF521* or *Zfp521* in human and murine hematopoietic progenitor cells, lead to an increase of the B-cell differentiation *in vitro*. Recently, it has also been showed that Ebf1 cooperates in a negative feedback loop to repress Zfp521 as differentiation proceeds, acting as a commitment switch between the adipogenic and osteogenic lineages [60]. Studies on the role of the transcription factor IKAROS during the control of early B-cell development, indicate that Zfp521 is among the repressed targets of IKAROS in pro-B cells [61]. ZNF521 has also been implicated in the development of B-lymphoid malignancies. A chromosomal aberration involving *ZNF521* is found in acute lymphoblastic leukemia, where *ZNF521* translocates with *PAX5*. The translocation generates the *PAX5-ZNF521* oncogene consisting of the N-terminus part of *PAX5* and the C-terminus part of *ZNF521* [62].

Mathematical modeling of transcriptional regulatory networks

The ability of specific proteins (transcription factors) to affect the transcription rate allows, for a network of proteins, to regulate each others production (or a network of genes regulating each others activity). The biological processes involved in transcription and translation are highly complex, and the associated mathematical descriptions are generally simplified approximations. This is most often sufficient due to the lack of detailed experimental data. It is also often convenient to model transcription and translation within a single equation, and due to the complex input-output relations for these processes, nonlinear descriptions are required. The transcription/translation process can be modeled as a transcription factor (TF) binding to DNA (creating a complex) which activates or represses the expression of a protein P. A model describing an activator is

$$DNA + TF \underset{k_1}{\overset{k_{1r}}{\rightleftharpoons}} DNA\colon TF \xrightarrow{k2} DNA\colon TF + P \tag{S1}$$

Assuming that the binding/release of the transcription factor is fast compared to the protein expression, it seems opportune applying a Michaelis-Menten formalism. The enzyme in this case is represented by DNA, and it can be assumed to exist as a single copy within a cell ($DNA + DNA\colon TF = 1$). Solving for the equilibrium of the left part of the reaction leads to $DNA = TF/(K + TF)$ where $K_a = k_{1r}/k_1$. This can be interpreted as the relative occupation of the binding site (or the fraction of time when TF is bound). The production of P can then be seen as this fraction times the rate of production when the regulation is active (given by $k_2 = V_{\max}$), which results in

$$\frac{dP}{dt} = V_{\max}\left(\frac{TF}{K + TF}\right) \tag{S2}$$

Note that in Michaelis-Menten transcription version, a problem with this formalism is the slow response to changes in substrate concentrations. For transcription this becomes even more evident, and a common extension of the Michaelis-Menten formalism is the Hill equation. The Hill-equation can be deduced from a model where a transcription factor can bind to DNA at multiple sites.

In the single transcription factor examples the rate limiting part of gene expression is typically the initiation of transcription. Modelling of one transcriptional factor activation are based on the assumption that the binding and unbinding of transcription factors were fast and could be assumed to be in equilibrium, which resulted in a probability for a bound and unbound state respectively. Then each of these states were connected to a rate for transcription. This idea can easily be extended to multiple transcription factors where the occupancy combined probabilities are used, [63]. The fractional occupancy of the operator site is then given by the degree of bound operator relative to the total of all occupancy states, that is

$$Y = \frac{\text{steady state level of activating states}}{\text{sum of the steady state levels of all DNA configurations}} \tag{S3}$$

where Y is the fractional DNA occupancy. For example, for two activators A, B, with a, b their related concentrations

$$Y_1 = \frac{k_0 + K_A a + K_B b + K_A a \cdot K_B b \cdot K_q}{1 + K_A a + K_B b + K_A a \cdot K_B b \cdot K_q} \tag{S4}$$

where k_0 is the basal expression rate, when no transcription factors act on the gene and K_q is a cooperativity factor ($K_q = 1$, if A and B bind independently). In case of one activator A and one repressor R (with r its concentration), the fractional occupancy is defined as follows

$$Y_2 = \frac{k_0 + K_A a}{1 + K_A a + K_R r + K_A a \cdot K_R r \cdot K_q} \tag{S5}$$

In case of one multimeric transcription factor, the fraction occupancy will assume Hill formalism

$$Y_3 = \frac{k_0 + (K_A a)^n}{1 + (K_A a)^n} \tag{S6}$$

Parameter estimation based on gene expression analysis

The parameter space has been, firstly, characterized adapting values consistent with microarray gene profiling of the CD19$^+$ CD127$^+$ and CD19$^+$ CD127$^-$ cells with B lineage cell subsets isolated from pediatric marrow reported in [64, 65]. The profiling was conducted using the Affymetrix platform Expression Console and the statistical analysis was performed considering Affymetrix Transcriptome Analysis Console (TAC) Software, S1 Fig.

Once the model is defined, it is possible to identify the conditions for which switch-like behavior can be elicited. At a glance, the parameter space related to the proposed model is high-dimensional space and searching for potentially interesting dynamics in this space is a non-trivial task. We are interested in qualitative changes in the model occurring in correspondence of particular points in the parameter space (bifurcations). From a purely control-theoretical approach, these points are characterized by particular changes in the eigenvalues that describe the local dynamics of the model. The Bifurcation Discovery Tool offers a possible approach to locating interesting qualitative properties in a model by means of an optimization method in order to search any bifurcation points, [66]. The tool is available at http://jdesigner.sourceforge.net/Site/Bifurcation_Discovery_Tool.html. A list of parameter values, in correspondence of which we found a set of parameter values that provide a classic bistable behavior for the proposed model, is given in S1 Table.

Irreversible bistable commitment switch between LMPPs and lymphoid LRPs

To reach the two steady states, we considered the following possible conditions: (*i*) suppression of EBF1 induced by reinforcing expression of ZNF521, or (*ii*) activation of B-cell development by reinforcing expression of EBF1. Quantitatively description of these conditions is modeled by including the external factors T_{EBF1}, T_{ZNF521}, which has the effect of enriching the expression of EBF1 and ZNF521, respectively. In S2a Fig, a time series is displayed for $T_{EBF1} = 0$ and $T_{ZNF521} = 0.2$, which shows the steady states being achieved from initial conditions corresponding to LMPP expression level, [64], such that multipotent progenitor specified genes are high and all other transcription factor concentrations are low. Therefore, reinforcing ZNF521 expression, by keeping low T_{EBF1} values, EBF1 is arrested in LMPP stage and the B lymphopoiesis lineage is switched off. When ZNF521 is not activated, T_{ZNF521} (no factor activates ZNF521 expression), the transcriptional regulatory network is able to turn on the B lymphopoiesis commitment, and the equilibrium between EBF1 and ZNF521 unbalances in favor of lymphoid LRPs specified genes, as EBF1, PAX5 and CD19, that which remain stable at higher levels, S2b Fig.

Sensitivity analysis of the bistable commitment switch

The quantification of the individual influence of the model parameters is the purpose to which is aimed a parametric sensitivity analysis, in order to understand the way how the nominal behavior of the system changes with respect to parameter variations. In this case, it has been considered to evaluate independently the effects of variations for each parameter with respect to the preservation of bistability. Looking at the dynamics

of the factor CD19, chosen to monitor the expression of B lymphocyte development, one can observe the effects of variations independently for each parameter on the system behavior.

All the parameters involved in the characterization of EBF1 and ZNF521 dynamics show bistability region in which, for the same parameter value, the system admits two stable points, S3 Fig. In this region, the system assumes the dynamics of a classical switch, able to guarantee the commitment of hematopoietic multipotent progenitors toward B lymphocyte precursors.

Varying independently the parameters involved in the dynamics of IL-7R and E2A factors, f_0, f_1, f_2, f_3 and j_0, j_1, respectively, the same behavior can be also observed, as expected, since they play a critical role in lymphopoiesis, and the encoded proteins are required for B lymphocyte development, S4 Fig. Notably, the parameter describing basal expression of FLT3 receptor, e_0, expressed on the surface of many hematopoietic progenitor cells, shows too a bistable regime for CD19 expression, S5 Fig.

Performing bifurcation study with respect to the remaining parameters, it can be verified that independent variations of each of these parameters are not able to show bistable region: in this case, for any variation CD19 is preserved in a single expression level, S5 Fig.

Inhibition of PAX5 on ZNF521 expression guarantees the commitment switching

In order to prove the crucial role of the inhibition of PAX5 on ZNF521 expression, described by b_4, on the basis of the proposed model (for the sake of clarity, we refer to model eqs. (1a-j) in the manuscript as Model A, we can evaluate other different configuration of the original transcriptional regulatory network, considering reasonable regulatory gene hypotheses.

Model B. Removing the direct inhibition of PAX5 on ZNF521 expression, let us suppose a direct repression of E2A on ZNF521 expression, b_{E2A}. This imply a variation only in eq. 1g in the original ODE model:

$$\begin{aligned}\frac{dx_{\mathrm{ZNF521}}}{dt} =&(b_0 + b_1 T_{ZNF521} + b_2 x_{\mathrm{ZNF521}})\\ &\times 1/(1 + b_1 T_{ZNF521} + b_2 x_{\mathrm{ZNF521}}\\ &+ b_3 x_{\mathrm{ZNF521}} x_{\mathrm{EBF1}}\\ &+ b_{E2A} x_{\mathrm{E2A}})\\ &- \mu_7 x_{\mathrm{ZNF521}}\end{aligned} \tag{S7}$$

Model C. Removing the direct inhibition of PAX5 on ZNF521 expression, let us consider a transcriptional repression of ZNF521 through IKAROS, as reported in [61]. This imply a variation in correspondence of eq. 1g in the original ODE model:

$$\begin{aligned}\frac{dx_{\mathrm{ZNF521}}}{dt} =&(b_0 + b_1 T_{ZNF521} + b_2 x_{\mathrm{ZNF521}})\\ &\times 1/(1 + b_1 T_{ZNF521} + b_2 x_{\mathrm{ZNF521}}\\ &+ b_3 x_{\mathrm{ZNF521}} x_{\mathrm{EBF1}}\\ &+ b_{IKA} x_{\mathrm{IKAROS}})\\ &- \mu_7 x_{\mathrm{ZNF521}}\end{aligned} \tag{S8}$$

References

1. Georgopoulos K, Bigby M, Wang JH, Molnar A, Wu P, Winandy S, et al. (1994) The Ikaros gene is required for the development of all lymphoid lineages. *Cell*, 79(1):143–156.

2. Wang JH, Nichogiannopoulou A, Wu L, Sun L, Sharpe AH, Bigby M, et al. (1996) Selective defects in the development of the fetal and adult lymphoid system in mice with an Ikaros null mutation. *Immunity*, 5(6):537–549.

3. Sellars ML, Kastner P, Chan S (2011) Ikaros in B-cell development and function. *World journal of biological chemistry*, 2(6):132.

4. Nutt SL, Kee BL (2007) The transcriptional regulation of B-cell lineage commitment. *Immunity*, 26(6):715–725.

5. Yoshida T, Ng SYM, Zuniga-Pflucker JC, Georgopoulos K (2006) Early hematopoietic lineage restrictions directed by Ikaros. *Nat Immunol*, 7(4):382–391.

6. Hesse JE, Lieber MR, Mizuuchi K, Gellert M (1989) V(D)J recombination: a functional definition of the joining signals. *Genes Dev*, 3(7):1053–1061.

7. Kirstetter P, Thomas M, Dierich A, Kastner P, Chan S (2002) Ikaros is critical for B-cell differentiation and function. *Eur J Immunol*, 32(3):720–730.

8. Ng SYM, Yoshida T, Georgopoulos K (2007) Ikaros and chromatin regulation in early hematopoiesis. *Curr Opin Immunol*, 19(2):116–122.

9. McKercher SR, Torbett BE, Anderson KL, Henkel GW, Vestal DJ, Baribault H, et al. (1996) Targeted disruption of the PU.1 gene results in multiple hematopoietic abnormalities. *EMBO J*, 15(20):5647–5658.

10. Valledor AF, Borràs FE, Cullell-Young M, Celada A (1998) Transcription factors that regulate monocyte/macrophage differentiation. *J Leukoc Biol*, 63(4):405–417.

11. Rekhtman N, Radparvar F, Evans T, Skoultchi A (1999) Direct interaction of hematopoietic transcription factors PU. 1 and GATA-1: functional antagonism in erythroid cells. *Genes & Development*, 13(11):1398–1411.

12. Zhang P, Zhang X, Iwama A, Yu C, Smith KA, Mueller BU, et al. (2000) PU.1 inhibits GATA-1 function and erythroid differentiation by blocking GATA-1 DNA binding. *Blood*, 96(8):2641–2684.

13. DeKoter RP, Singh H (2000) Regulation of B lymphocyte and macrophage development by graded expression of PU.1. *Science*, 288(5470):1439–1441.

14. Carotta S, Dakic A, D'Amico A, Pang SHM, Greig KT, Nutt SL, et al. (2010) The transcription factor PU.1 controls dendritic cell development and FLT3 cytokine receptor expression in a dose-dependent manner. *Immunity*, 32(5):628–641.

15. Okuno Y, Huang G, Rosenbauer F, Evans EK, Radomska HS, Iwasaki H, et al. (2005) Potential autoregulation of transcription factor PU.1 by an upstream regulatory element. *Mol Cell Biol*, 25(7):2832–2845.

16. DeKoter RP, Schweitzer BL, Kamath MB, Jones D, Tagoh H, Bonifer C, et al. (2007) Regulation of the interleukin-7 receptor alpha promoter by the Ets transcription factors PU.1 and GA-binding protein in developing B-cells. *J Biol Chem*, 282(19):14194–14204.

17. Spooner CJ, Cheng JX, Pujadas E, Laslo P, Singh H (2009) A recurrent network involving the transcription factors PU.1 and Gfi1 orchestrates innate and adaptive immune cell fates. *Immunity*, 31(4):576–586.

18. De Koter RP, Lee HJ, Singh H (2002) PU.1 regulates expression of the interleukin-7 receptor in lymphoid progenitors. *Immunity*, 16(2):297–309.

19. Dakic A, Metcalf D, Di Rago L, Mifsud S, Wu L, Nutt SL (2005) PU.1 regulates the commitment of adult hematopoietic progenitors and restricts granulopoiesis. *J Exp Med*, 201(9):1487–1502.

20. Adolfsson J, Borge OJ, Bryder D, Theilgaard-Mönch K, Astrand-Grundström I, Sitnicka E, et al. (2001) Upregulation of Flt3 expression within the bone marrow Lin(-)Sca1(+)c-kit(+) stem cell compartment is accompanied by loss of self-renewal capacity. *Immunity*, 15(4):659–669.

21. Mackarehtschian K, Hardin JD, Moore KA, Boast S, Goff SP, Lemischka IR (1995) Targeted disruption of the flk2/flt3 gene leads to deficiencies in primitive hematopoietic progenitors. *Immunity*, 3(1):147–161.

22. Li LX, Goetz CA, Katerndahl CDS, Sakaguchi N, Farrar MA (2010) A Flt3- and Ras-dependent pathway primes B-cell development by inducing a state of IL-7 responsiveness. *J Immunol*, 184(4):1728–1736.

23. Kroemer RT, Richards WG (1996) Homology modeling study of the human interleukin-7 receptor complex. *Protein Eng*, 9(12):1135–1142.

24. Miller JP, Izon D, DeMuth W, Gerstein R, Bhandoola A, Allman D (2002) The earliest step in B lineage differentiation from common lymphoid progenitors is critically dependent upon interleukin 7. *J Exp Med*, 196(5):705–711.

25. Corfe SA, Paige CJ (2012) The many roles of IL-7 in B-cell development; mediator of survival proliferation and differentiation. *Semin Immunol*, 24(3):198–208.

26. Sitnicka E, Brakebusch C, Martensson IL, Svensson M, Agace WW, Sigvardsson M, et al. (2003) Complementary signaling through flt3 and interleukin-7 receptor alpha is indispensable for fetal and adult B-cell genesis. *J Exp Med*, 198(10):1495–1506.

27. Peschon JJ, Morrissey PJ, Grabstein KH, Ramsdell FJ, Maraskovsky E, Gliniak BC, et al. (1994) Early lymphocyte expansion is severely impaired in interleukin 7 receptor-deficient mice. *J Exp Med*, 180(5):1955–1960.

28. Schebesta M, Heavey B, Busslinger M (2002) Transcriptional control of B-cell development *Curr Opin Immunol*, 14(2):216–223.

29. Dias S, Månsson R, Gurbuxani S, Sigvardsson M, Kee BL (2008) E2A proteins promote development of lymphoid-primed multipotent progenitors. *Immunity*, 29(2):217–227.

30. Kwon K, Hutter C, Sun Q, Bilic I, Cobaleda C, Malin S, et al. (2008) Instructive role of the transcription factor E2A in early B lymphopoiesis and germinal center B-cell development. *Immunity*, 28(6):751–762.

31. Borghesi L, Aites J, Nelson S, Lefterov P, James P, Gerstein R (2005) E47 is required for V(D)J recombinase activity in common lymphoid progenitors. *J Exp Med*, 202(12):1669–1677.

32. Welinder E, Månsson R, Mercer EM, Bryder D, Sigvardsson M, Murre C (2011) The transcription factors E2A and HEB act in concert to induce the expression of FOXO1 in the common lymphoid progenitor. *Proc Natl Acad Sci USA*, 108(42):17402–17407.

33. Chaudhary J, Skinner MK (1999) Basic helix-loop-helix proteins can act at the E-box within the serum response element of the c-fos promoter to influence hormone-induced promoter activation in Sertoli cells. *Mol Endocrinol*, 13(5):774–786.

34. Smith KS, Rhee JW, Cleary ML (2002) Transformation of bone marrow B-cell progenitors by E2a-Hlf requires coexpression of Bcl-2. *Mol Cell Biol*, 22(21):7678–7687.

35. Medina KL, Pongubala JMR, Reddy KL, Lancki DW, Dekoter R, Kieslinger M, et al. (2004) Assembling a gene regulatory network for specification of the B-cell fate. *Dev Cell*, 7(4):607–617.

36. Lin H, Grosschedl R (1995) Failure of B-cell differentiation in mice lacking the transcription factor EBF. *Nature*, 376(6537):263–267.

37. Sigvardsson M, O'Riordan M, Grosschedl R (1997) EBF and E47 collaborate to induce expression of the endogenous immunoglobulin surrogate light chain genes. *Immunity*, 7(1):25–36.

38. Dias S, Silva JrH, Cumano A, Vieira P (2005) Interleukin-7 is necessary to maintain the B-cell potential in common lymphoid progenitors. *J Exp Med*, 201(6):971–979.

39. Kikuchi K, Lai AY, Hsu CL, Kondo M (2005) IL-7 receptor signaling is necessary for stage transition in adult B-cell development through up-regulation of EBF. *J Exp Med*, 201(8):1197–1203.

40. Seet CS, Brumbaugh RL, Kee BL (2004) Early B-cell factor promotes B lymphopoiesis with reduced interleukin 7 responsiveness in the absence of E2A. *J Exp Med*, 199(12):1689–1700.

41. Pongubala JMR, Northrup DL, Lancki DW, Medina KL, Treiber T, Bertolino E, et al. (2008) Transcription factor EBF restricts alternative lineage options and promotes B-cell fate commitment independently of Pax5. *Nat Immunol*, 9(2):203–215.

42. Roessler S, Györy I, Imhof S, Spivakov M, Williams RR, Busslinger M, et al. (2007) Distinct promoters mediate the regulation of Ebf1 gene expression by interleukin-7 and Pax5. *Mol Cell Biol*, 27(2):579–594.

43. Zandi S, Månsson R, Tsapogas P, Zetterblad J, Bryder D, Sigvardsson M (2008) EBF1 is essential for B-lineage priming and establishment of a transcription factor network in common lymphoid progenitors. *J Immunol*, 181(5):3364–3372.

44. Decker T, Pasca di Magliano M, McManus S, Sun Q, Bonifer C, Tagoh H, et al. (2009) Stepwise activation of enhancer and promoter regions of the B-cell commitment gene Pax5 in early lymphopoiesis. *Immunity*, 30(4):508–520.

45. Fuxa M, Busslinger M (2007) Reporter gene insertions reveal a strictly B lymphoid-specific expression pattern of Pax5 in support of its B-cell identity function. *J Immunol*, 178(12):8222–8228.

46. Pridans C, Holmes ML, Polli M, Wettenhall JM, Dakic A, Corcoran LM, et al. (2008) Identification of Pax5 target genes in early B-cell differentiation. *J Immunol*, 180(3):1719–1728.

47. Kozmik Z, Wang S, Dörfler P, Adams B, Busslinger M (1992) The promoter of the CD19 gene is a target for the B-cell-specific transcription factor BSAP. *Mol Cell Biol*, 12(6):2662–2672.

48. Medvedovic J, Ebert A, Tagoh H, Busslinger M (2011) Pax5: a master regulator of B-cell development and leukemogenesis. *Adv Immunol*, 111:179–206.

49. Holmes ML, Carotta S, Corcoran LM, Nutt SL (2006) Repression of Flt3 by Pax5 is crucial for B-cell lineage commitment. *Genes Dev*, 20(8):933–938.

50. Mullighan CG, Goorha S, Radtke I, Miller CB, Coustan-Smith E, Dalton JD, et al. (2007) Genome-wide analysis of genetic alterations in acute lymphoblastic leukaemia. *Nature*, 446: 758–764.

51. Hardy RR, Kincade PW, Dorshkind K (2007) The protean nature of cells in the B lymphocyte lineage. *Immunity*, 26(6):703–714.

52. Lukin K, Fields S, Hartley J, Hagman J (2008) Early B-cell factor: Regulator of B lineage specification and commitment. *Semin Immunol*, 20(4):221–227.

53. Mega T, Lupia M, Amodio N, Horton SJ, Mesuraca M, Pelaggi D, et al. (2011) Zinc finger protein 521 antagonizes early B-cell factor 1 and modulates the B lymphoid differentiation of primary hematopoietic progenitors. *Cell Cycle*, 10(13):2129–2139.

54. Gerrits A (2011) in *Exploiting Natural and Induced Genetic Variation to Study Hematopoiesis*, PhD Thesis, University Library of Groningen.

55. Warming S, Liu P, Suzuki T, Akagi K, Lindtner S, Pavlakis GN, et al. (2003) Evi3, a common retroviral integration site in murine B-cell lymphoma, encodes an EBFAZ-related Krüppel-like zinc finger protein. *Blood*, 101(5):1934–1940.

56. Bond HM, Mesuraca M, Carbone E, Bonelli P, Agosti V, Amodio N, et al. (2004) Early hematopoietic zinc finger protein (EHZF), the human homolog to mouse Evi3, is highly expressed in primitive human hematopoieticv ells. *Blood*, 103(6):2062–2070.

57. Kamiya D, Banno S, Sasai N, Ohgushi M, Inomata H, Watanabe K, et al. (2011) Intrinsic transition of embryonic stem-cell differentiation into neural progenitors. *Nature*, 470(7335):503–509.

58. Wu M, Hesse E, Morvan F, Zhang JP, Correa D, Rowe GC, et al. (2009) Zfp521 antagonizes Runx2, delays osteoblast differentiation in vitro, and promotes bone formation in vivo. *Bone*, 44(4):528–536.

59. Lobo MK, Yeh C, Yang XW (2008) Pivotal role of early B-cell factor 1 in development of striatonigral medium spiny neurons in the matrix compartment. *J Neurosci Res*, 86(10):2134–2146.

60. Kang S, Akerblad P, Kiviranta R, Gupta RK, Kajimura S, Griffin MJ, et al. (2012) Regulation of early adipose commitment by Zfp521. *PLoS Biol*, 10(11):e1001433.

61. Schwickert TA, Tagoh H, Gultekin S, Dakic A, Axelsson E, Minnich M, et al. (2014) Stage-specific control of early B cell development by the transcription factor Ikaros. *Nat Immunol*, 15:283-293.

62. Nebral K, König M, Harder L, Siebert R, Haas OA, Strehl S (2007) Identification of PML as novel PAX5 fusion partner in childhood acute lymphoblastic leukaemia. *Br J Haematol*, 139(2):269–274.

63. Sauro HM (2012) in *Enzyme Kinetics for Systems Biology*, eds. Future Skill Software (Ambrosius Publishing), pp.203–248.

64. van Zelm MC, van der Burg M, de Ridder D, Barendregt BH, de Haas EFE, Reinders MJT, et al. (2005) Ig gene rearrangement steps are initiated in early human precursor B-cell subsets and correlate with specific transcription factor expression. *J Immunol*, 175(9):5912–5922.

65. Nodland SE, Berkowska MA, Bajer AA, Shah N, de Ridder D, van Dongen JJM, et al. (2011) IL-7R expression and IL-7 signaling confer a distinct phenotype on developing human B-lineage cells. *Blood*, 118(8):2116–2127.

66. Chickarmane V, Paladugu SR, Bergmann F, Sauro HM (2005) Bifurcation discovery tool. *Bioinformatics*, 21(18):3688–3690.

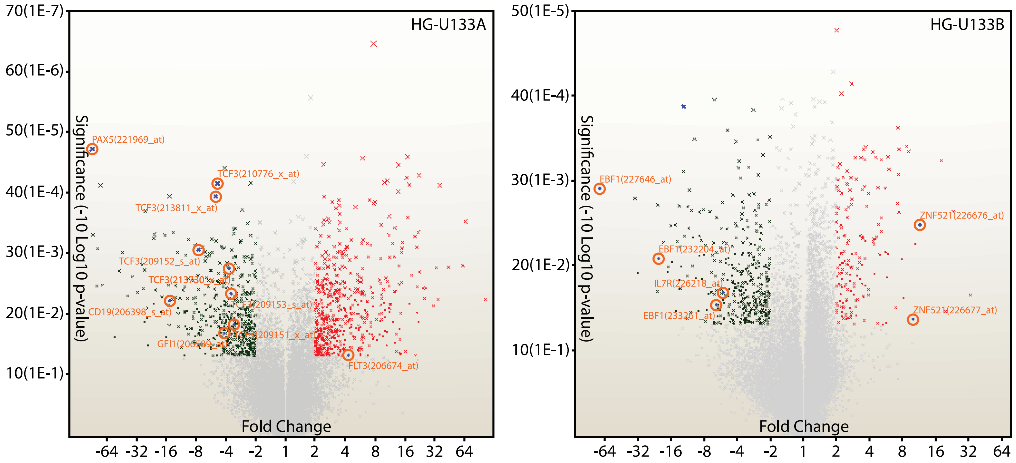

Figure S1. Volcano plot showing gene level differential expression between the two conditions CD34$^+$/lin$^-$ and pro-B vs. significance value.(a) When the ZNF521 is activated, the LMPPs specified genes are highly expressed arresting the development of B cells. Differently,**(b)** when no factor stimulates the expression of ZNF521, the system is driven towards the lymphoid lineage fate, resulting in a higher expression of the B-lymphoid LRP specified genes.

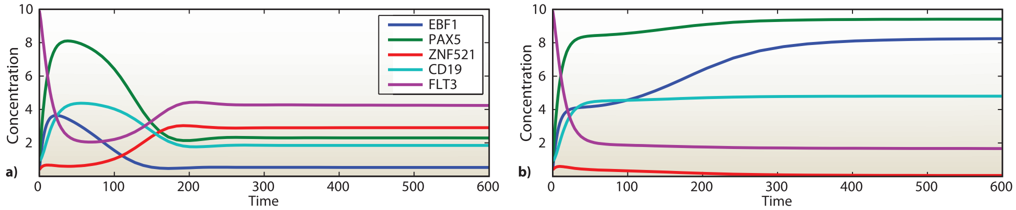

Figure S2. Time profile concentrations of FLT3, ZNF521, EBF1, PAX5 and CD19 for the two cellular stages, indicating the final steady state values. (a) When the ZNF521 is activated, the LMPPs specified genes are highly expressed arresting the development of B cells. Differently, **(b)** when no factor stimulates the expression of ZNF521, the system is driven towards the lymphoid lineage fate, resulting in a higher expression of the B-lymphoid LRP specified genes.

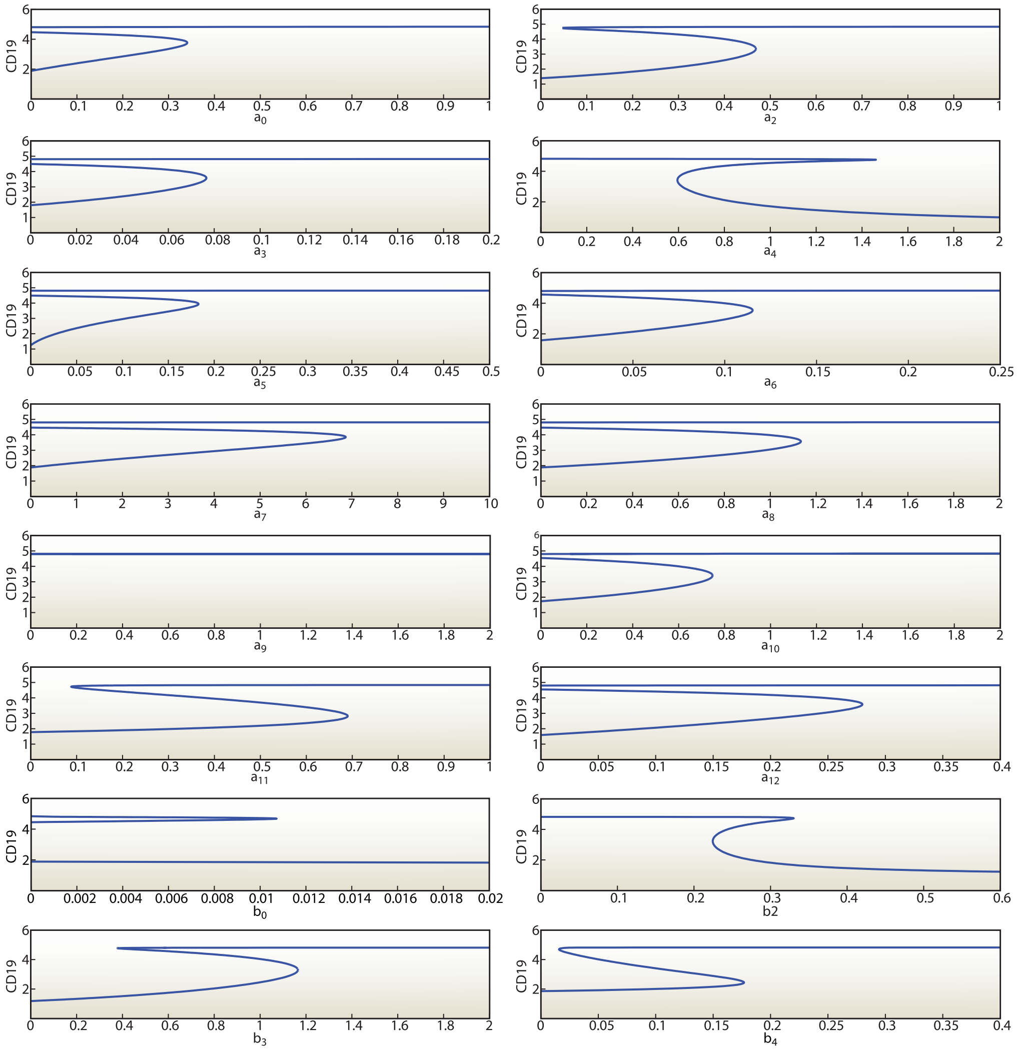

Figure S3. CD19 bifurcation analysis with respect to the parameters involved in EBF1 and ZNF521 transcriptional dynamics. The parameters associated to EBF1 transcription, $a_0, a_2, \ldots, a_8, a_{10}, \ldots, a_{12}$, and all the parameters involved in ZNF521 transcription, $b_0, b_2, \ldots, b_4$ exhibit an interval where bistable solutions can be found.

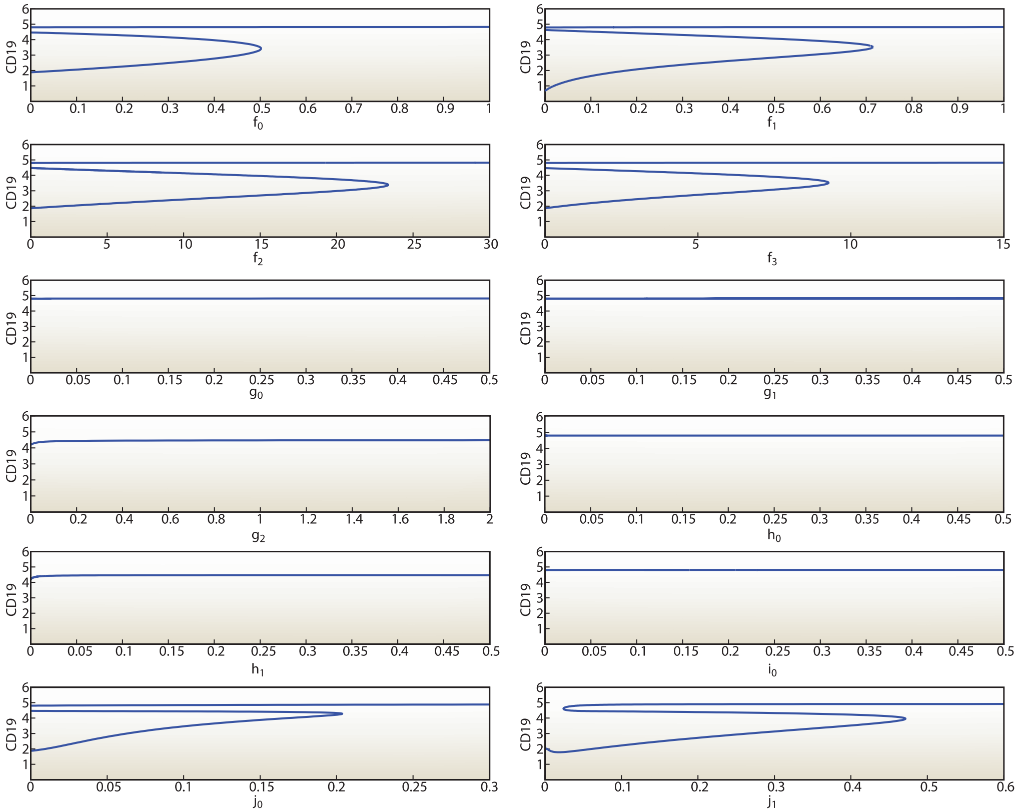

Figure S4. CD19 bifurcation analysis with respect to the parameters involved in IL-7R, PU.1, IKAROS and E2A transcriptional dynamics. The parameters associated to IL-7R transcription, $f_0, \ldots, f_3$ and to E2A transcription, j_0, j_1 exhibit an interval where bistable solutions can be found. Individual variations of the kinetic parameters describing the PU.1 dynamics, $g_0, \ldots, g_2$, the GFI1 dynamics, h_0, h_1 and the basal expression of IKAROS, i_0, instead, do not give rise to bistability.

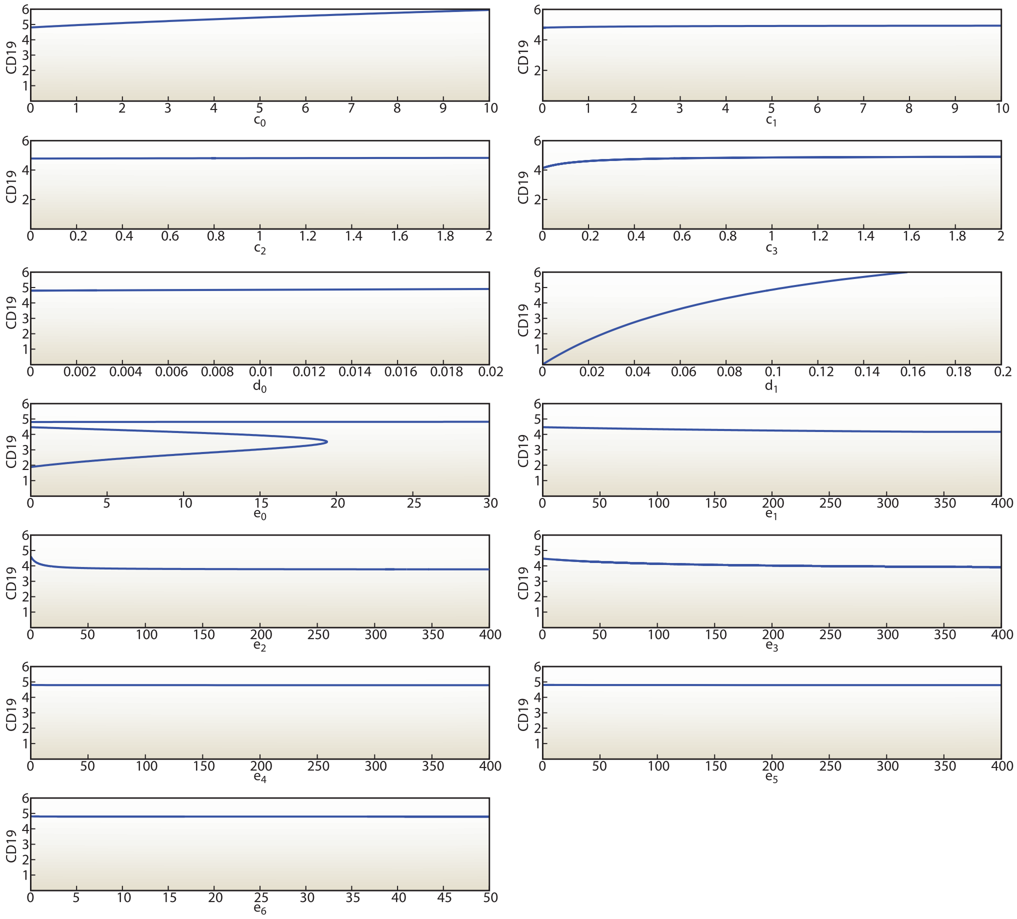

Figure S5. CD19 bifurcation analysis with respect to the parameters involved in PAX5, CD19 and FLT3 transcriptional dynamics. The parameters associated to PAX5 transcription, $c_0, \ldots, c_3$, to CD19 transcription d_0, d_1 and all the parameters involved in FLT3 transcription, $e_1, \ldots, e_6$ (with the only exception of its basal expression, e_0), do not give rise to bistability.

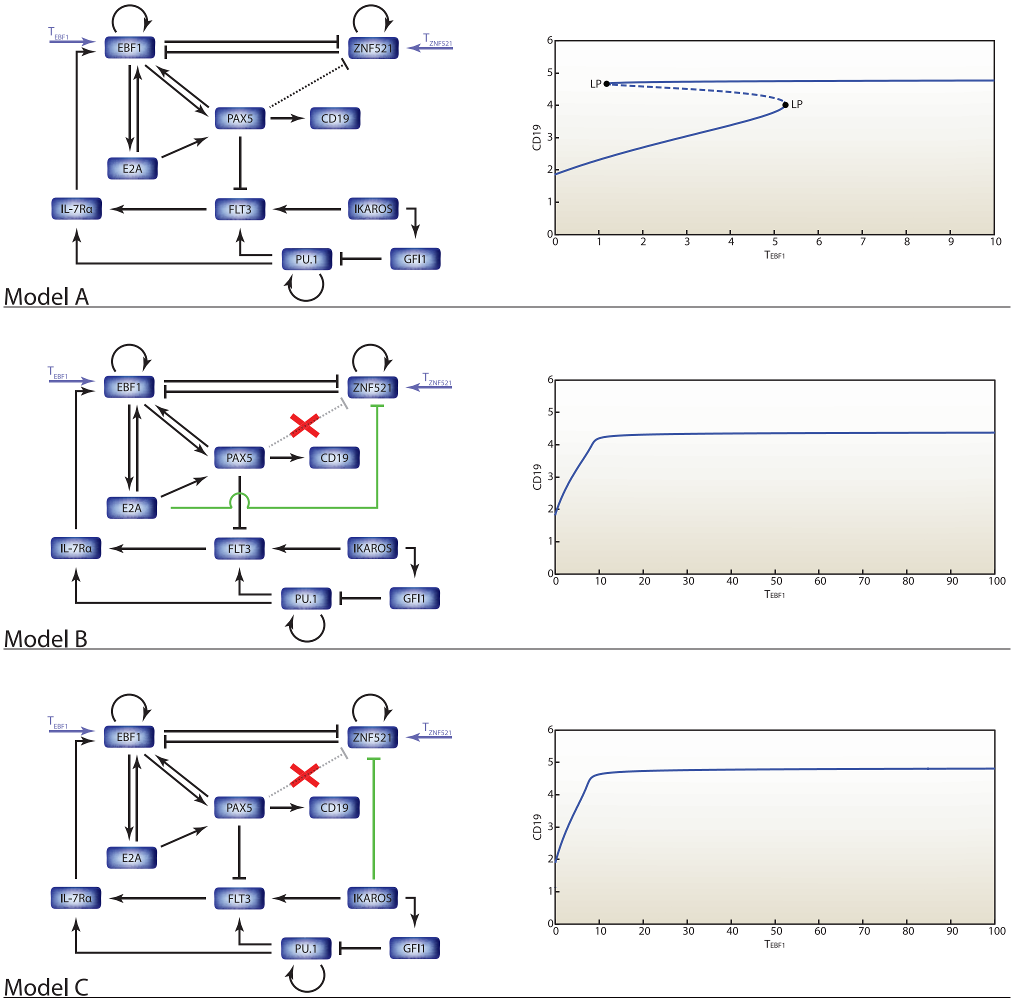

Figure S6. Comparison of the proposed model with different hypothetical configurations. Bifurcation study proves the crucial role of the inhibition of PAX5 on ZNF521 expression to admit the existence of commiment switching between LMPP and pro-B stages in B-lymphocyte development. Starting numerical continuation from the LMPP stage, let us impose the conditions related to the case of reversible commitment switch, for $T_{ZNF521} = 0.12$. **Model A.** As already seen, ZNF521 inhibition through PAX5 generates a bistable commitment switch between LMPPs (lower values of CD19 expression) and LRP cells (greater values of CD19 expression). **Model B.** Removing ZNF521 inhibition through PAX5, the effects of inhibition of ZNF521 through the factor E2A have been considered. In this case, the system is characterized by a single CD19 expression level, preventing the occurrence of switching behavior. **Model C.** Removing ZNF521 inhibition through PAX5, the effects of ZNF521 repression through IKAROS have been considered. Also in this case, CD19 reaches a single expression level.

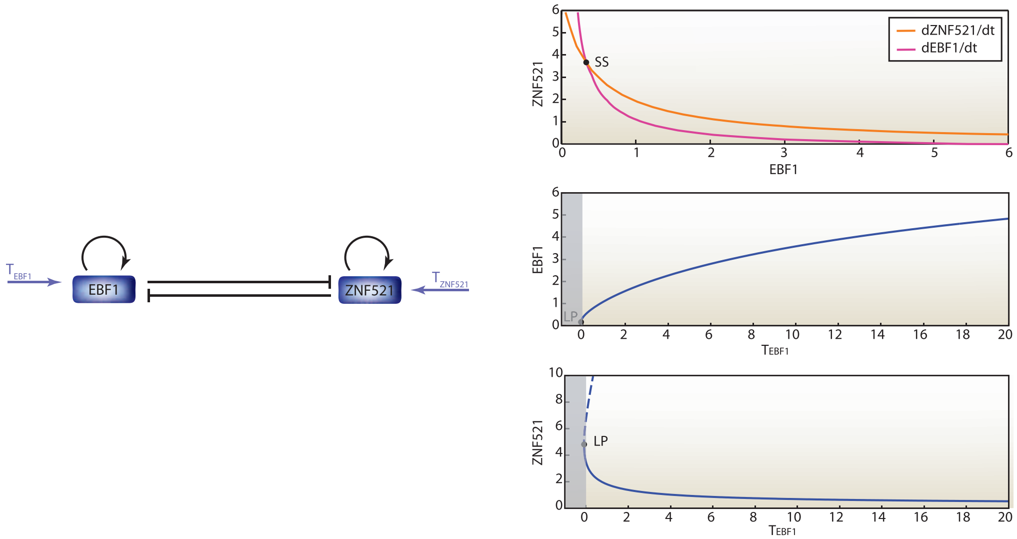

Figure S7. Dynamical analysis of the EBF1/ZNF521 feedback module. The EBF1-ZNF521 mutual inhibition, with autoregulatory interactions; the environmental factors, which promote the transcriptional activity of EBF1 and ZNF521 are indicated as T_{EBF1} and T_{ZNF521}, respectively. The intersection among the nullclines $d\text{ZNF521}/dt = 0$ and $d\text{EBF1}/dt = 0$, with parameters given by S1 Table, identifies a single stable point (SS) corresponding to the LMPP point (upper right panel). Bifurcation analysis performed promoting the transcriptional activity of EBF1 shows the existence of a single inaccessible turning point (LP), for negative values of T_{EBF1} (lower right panels). Solid line denotes stable steady-states, dashed line denotes unstable ones.

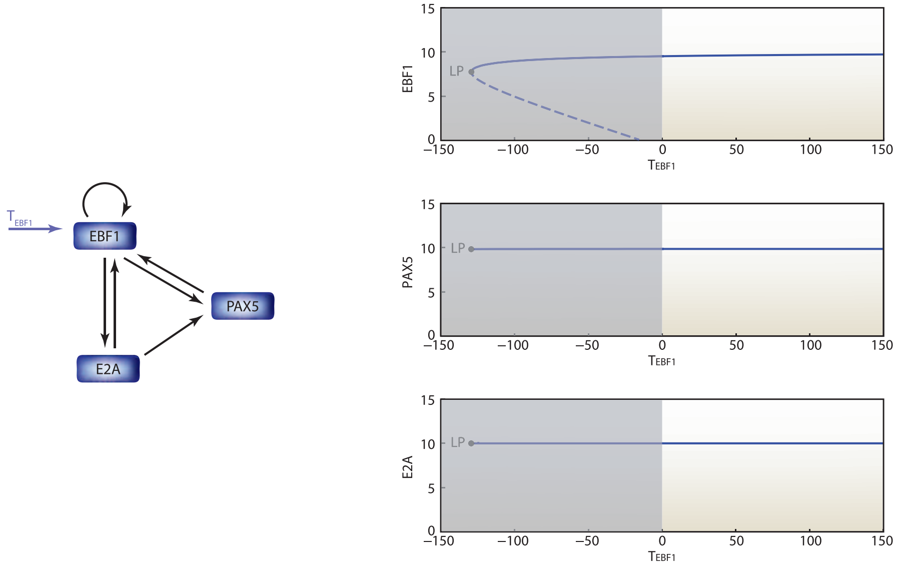

Figure S8. Dynamical analysis of the EBF1/E2A/PAX5 feedback module. The positive feedback loop constituted by EBF1, E2A and PAX5, with autoregulatory loop and the environmental factor, T_{EBF1}, acting on EBF1 transcription. Bifurcation analysis shows the expression levels of the corresponding factor as functions of T_{EBF1}. It is possible to note that this feedback sustains the committed state, admitting only one stable positive point (right panels). Solid line denotes stable steady-states, dashed line denotes unstable ones.

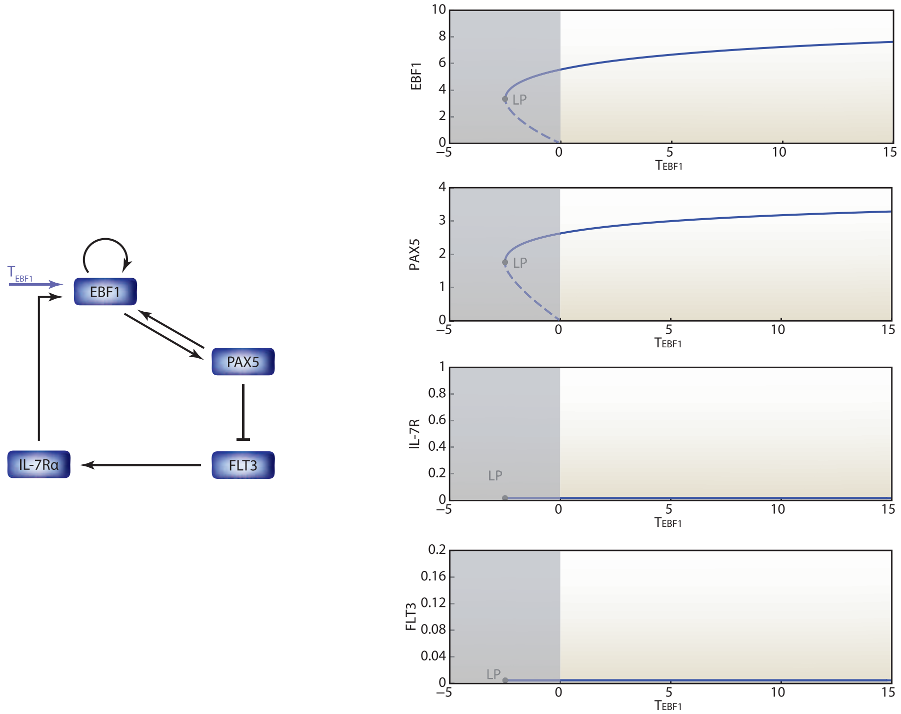

Figure S9. Dynamical analysis of the EBF1/PAX5/FLT3/IL-7R feedback module. As suggested by experimental findings, EBF1, PAX5, FLT3 and IL-7R form a negative loop (left panel). Bifurcation analysis performed with respect to T_{EBF1} values shows that this feedback admits a single positive steady state, relative to the committed state, in correspondence of which the expression of EBF1 and PAX5 are sustained at higher level and the FLT3 and IL-7R at lower ones (right panels). Solid line denotes stable steady-states, dashed line denotes unstable ones.

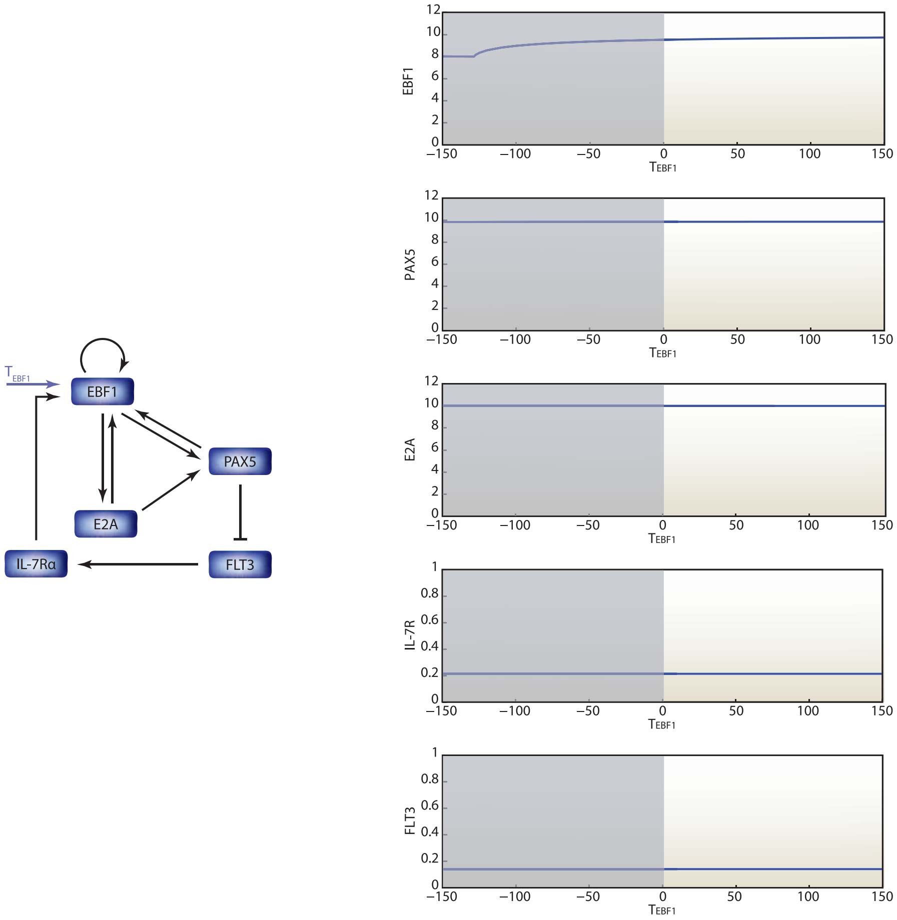

Figure S10. Dynamical analysis of the EBF1/E2A/PAX5/FLT3/IL-7R feedback module. The feedback loop comprising the positive loop involving EBF1, E2A and PAX5 and the negative one constituted by EBF1, PAX5, FLT3 and IL-7R (left panel). Bifurcation diagrams show the expression levels of the involved factors as functions of T_{EBF1}. It is possible to observe a reinforcement of the committed state, which does not provide the sought-after switch-like behavior (right panels).

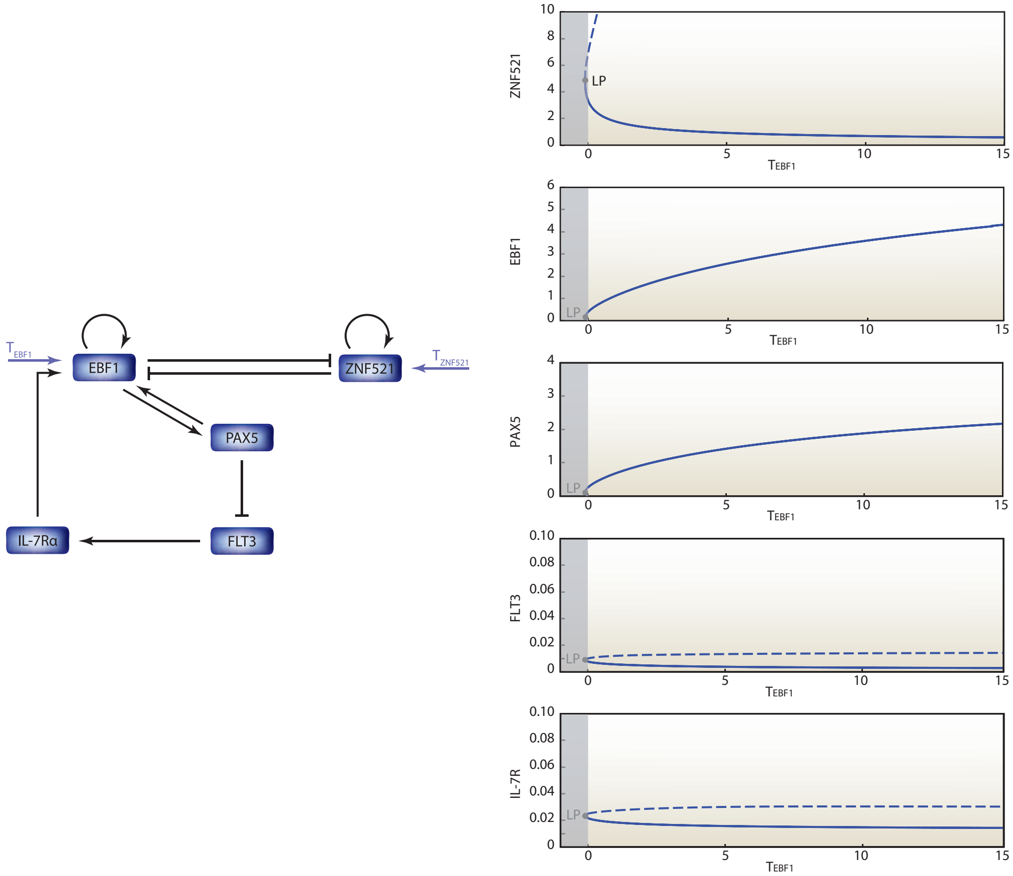

Figure S11. Dynamical analysis of the ZNF521/EBF1/PAX5/FLT3/IL-7R feedback module. The module constituted by the mutual inhibition between ZBF521 and EBF1, with autoregulatory loops, and the negative feedback formed by EBF1/PAX5/FLT3/IL-7R (left panel), does not appear to be sufficient to bistability behavior. Bifurcation analysis performed versus T_{EBF1} show the existence of only positive steady-states relative to the committed lineage (right panels). Solid line denotes stable steady-states, dashed line denotes unstable ones.

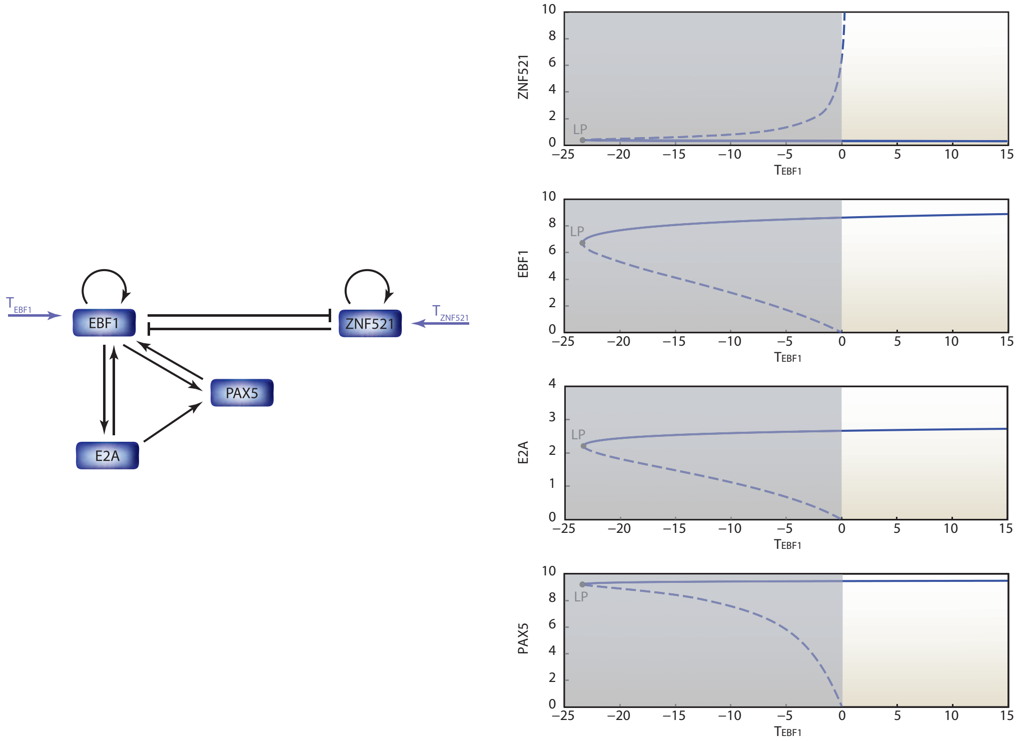

Figure S12. Dynamical analysis of the ZNF521/EBF1/PAX5/E2A feedback module. The module constituted by the mutual inhibition between ZBF521 and EBF1, with autoregulatory loops, and the positive feedback loop that sustain the committed state, EBF1/E2A/PAX5 (left panel), are unable to capture the required switch-like behavior. Observing the bifurcation diagrams (right panels) for consistent values of T_{EBF1} the system converges to the committed state. Solid line denotes stable steady-states, dashed line denotes unstable ones.

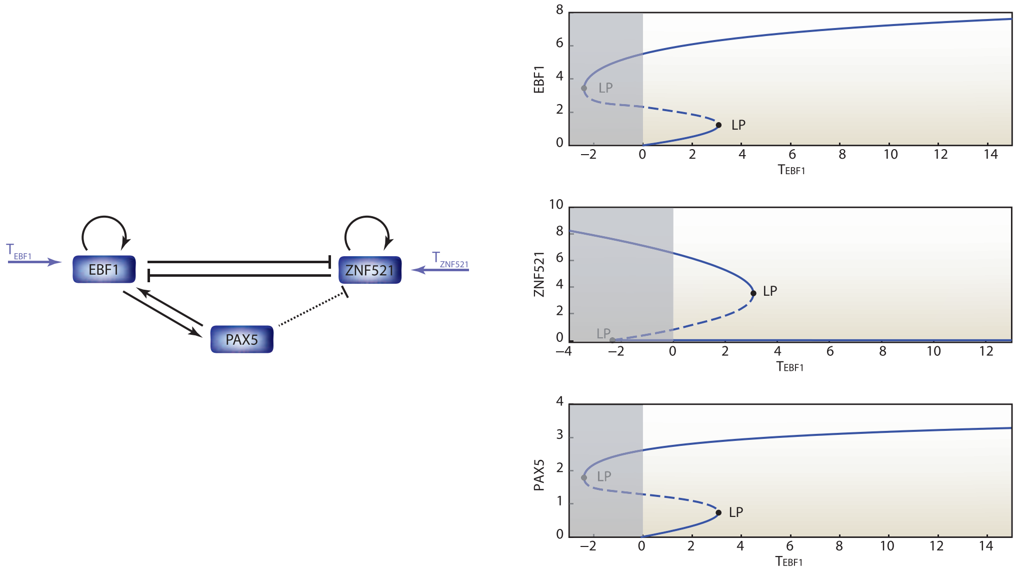

Figure S13. Dynamical analysis of the EBF1/PAX5/ZNF521 feedback module. The EBF1/ZNF521 mutual inhibition, with autoregulatory loops, is enriched by a further interaction through the key factor PAX5. Observing the bifurcation diagrams (right panels) with respect to the T_{EBF1} factor, this feedback induces irreversible bistable behavior. Solid line denotes stable steady-states, dashed line denotes unstable ones.

Table S1. Parameter values used in the dynamical equations.

Parameter	Value	Description
a_0	0.001	basal transcription rate for EBF1
a_1	0.1	environmental factor promoting EBF1 transcription
a_2	0.22	parameters involved in the transcriptional activity of EBF1
a_3	0.007	
a_4	0.9	
a_5	0.022	
a_6	0.029	
a_7	0.005	
a_8	0.00022	
a_9	0.19	
a_{10}	0.135	
a_{11}	0.171	
a_{12}	0.064	
b_0	0.001	basal transcription rate for ZNF521
b_1	0.1	
b_2	0.29	Parameters involved in the transcriptional activity of ZNF521
b_3	0.7	
b_4	0.023	
c_0	0.001	basal transcription rate for PAX5
c_1	0.064	parameters involved in the transcriptional activity of PAX5
c_2	0.82	
c_3	0.63	
d_0	0.001	basal transcription rate for CD19
d_1	0.098	parameters involved in the transcriptional activity of CD19
μ_1	0.0025	degradation rate for IKAROS
e_0	0.001	basal transcription rate for FLT3
e_1	1.33	parameters involved in the transcriptional activity of FLT3
e_2	1.26	
e_3	0.69	
e_4	1.15	
e_5	0.12	
e_6	0.4	
f_0	0.001	basal transcription rate for IL-7R
f_1	0.15	parameters involved in the transcriptional activity of IL-7R
f_2	0.27	
f_3	0.069	
g_0	0.001	basal transcription rate for PU.1
g_1	0.11	parameters involved in the transcriptional activity of PU.1
g_2	0.86	
h_0	0.001	basal transcription rate for GFI1
h_1	0.235	parameters involved in the transcriptional activity of GFI1
i_0	0.01	basal transcription rate for IKAROS
j_0	0.001	basal transcription rate for E2A
j_1	0.042	parameters involved in the transcriptional activity of E2A
$\mu_{2,\ldots,10}$	0.1	degradation rates

Set of parameter values in correspondence of which the main model exhibits a classic bistable behavior.

Table S2. Parameter sensitivity coefficients for bistability.

Param	Min value	Max value	Sensitivity, FC*	Factor regulated
a_0	< 0	0.341	-inf; 8.414	param. controlling EBF1 dynamics
a_2	0.044	0.4691	-2.319; 1.092	
a_3	< 0	0.077	-inf; 3.459	
a_4	0.595	1.461	-0.597; 0.699	
a_5	< 0	0.183	-inf; 3.056	
a_6	< 0	0.115	-inf; 1.988	
a_7	< 0	6.868	-inf; 10.424	
a_8	< 0	1.134	-inf; 12.332	
a_{10}	< 0	0.748	-inf; 2.470	
a_{11}	0.088	0.690	-inf; 2.013	
a_{12}	< 0	0.280	-inf; 4.375	
b_0	< 0	0.011	-inf; 3.459	param. controlling ZNF521 dynamics
b_2	0.224	0.330	-0.373; 0.186	
b_3	0.381	1.165	-0.878; 0.735	
b_4	0.016	0.177	-0.524; 2.944	
e_0	< 0	19.380	-inf; 14.242	param. controlling FLT3 dynamics
f_0	< 0	0.502	-inf; 8.972	param. controlling IL-7R dynamics
f_1	< 0	0.715	-inf; 2.253	
f_2	< 0	22.380	-inf; 6.373	
f_3	< 0	9.267	-inf; 7.069	
j_0	< 0	0.204	-inf; 7.672	param. controlling E2A dynamics
j_1	0.024	0.471	-0.807; 3.487	

* The parameter sensitivity for bistability is defined as the log2 fold change of the ratio between the max (min) parameter value for which the system is bistable and the parameter used in the model (S1 Table).

Table S3. Parameter values used in the model B and C bifurcation studies.

Parameter	Value	Model	Description
b_{e2a}	0.03	B	Inhibition of ZNF521 through E2A
b_{ika}	0.25	C	Inhibition of ZNF521 through IKAROS
b_4	0.0	$ModelB, C$	Removal of ZNF521 inhibition through PAX5

Parameters values used as different configurations of the original transcriptional regulatory network, considering reasonable regulatory gene hypotheses.
