# Supplementary material for: Computational Modeling of a Transcriptional Switch Underlying B-Lymphocyte Lineage Commitment of Hematopoietic Multipotent Cells
Source: PLoS One. 2015 Jul 13;10(7):e0132208. doi: 10.1371/journal.pone.0132208 (PMC4500571; doi:10.1371/journal.pone.0132208)
Supplement: S1 Table — Set of parameter values in correspondence of which the main model exhibits a classic bistable behavior. (PDF) [file pone.0132208.s015.pdf]

Table S1. Parameter values used in the dynamical equations.

Parameter	Value	Description
a_0	0.001	basal transcription rate for EBF1
a_1	0.1	environmental factor promoting EBF1 transcription
a_2	0.22	parameters involved in the transcriptional activity of EBF1
a_3	0.007	
a_4	0.9	
a_5	0.022	
a_6	0.029	
a_7	0.005	
a_8	0.00022	
a_9	0.19	
a_{10}	0.135	
a_{11}	0.171	
a_{12}	0.064	
b_0	0.001	basal transcription rate for ZNF521
b_1	0.1	
b_2	0.29	Parameters involved in the transcriptional activity of ZNF521
b_3	0.7	
b_4	0.023	
c_0	0.001	basal transcription rate for PAX5
c_1	0.064	parameters involved in the transcriptional activity of PAX5
c_2	0.82	
c_3	0.63	
d_0	0.001	basal transcription rate for CD19
d_1	0.098	parameters involved in the transcriptional activity of CD19
μ_1	0.0025	degradation rate for IKAROS
e_0	0.001	basal transcription rate for FLT3
e_1	1.33	parameters involved in the transcriptional activity of FLT3
e_2	1.26	
e_3	0.69	
e_4	1.15	
e_5	0.12	
e_6	0.4	
f_0	0.001	basal transcription rate for IL-7R
f_1	0.15	parameters involved in the transcriptional activity of IL-7R
f_2	0.27	
f_3	0.069	
g_0	0.001	basal transcription rate for PU.1
g_1	0.11	parameters involved in the transcriptional activity of PU.1
g_2	0.86	
h_0	0.001	basal transcription rate for GFI1
h_1	0.235	parameters involved in the transcriptional activity of GFI1
i_0	0.01	basal transcription rate for IKAROS
j_0	0.001	basal transcription rate for E2A
j_1	0.042	parameters involved in the transcriptional activity of E2A
$\mu_{2,\ldots,10}$	0.1	degradation rates

Set of parameter values in correspondence of which the main model exhibits a classic bistable behavior.
