# Supplementary material for: Computational Modeling of a Transcriptional Switch Underlying B-Lymphocyte Lineage Commitment of Hematopoietic Multipotent Cells
Source: PLoS One. 2015 Jul 13;10(7):e0132208. doi: 10.1371/journal.pone.0132208 (PMC4500571; doi:10.1371/journal.pone.0132208)
Supplement: S2 Table — The parameter sensitivity for bistability is defined as the log2 fold change of the ratio between the max (min) parameter value for which the system is bistable and the parameter used in the model, S1 Table. (PDF) [file pone.0132208.s016.pdf]

Table S2. Parameter sensitivity coefficients for bistability.

Param	Min value	Max value	Sensitivity, FC*	Factor regulated
a_0	< 0	0.341	-inf; 8.414	param. controlling EBF1 dynamics
a_2	0.044	0.4691	-2.319; 1.092	
a_3	< 0	0.077	-inf; 3.459	
a_4	0.595	1.461	-0.597; 0.699	
a_5	< 0	0.183	-inf; 3.056	
a_6	< 0	0.115	-inf; 1.988	
a_7	< 0	6.868	-inf; 10.424	
a_8	< 0	1.134	-inf; 12.332	
a_{10}	< 0	0.748	-inf; 2.470	
a_{11}	0.088	0.690	-inf; 2.013	
a_{12}	< 0	0.280	-inf; 4.375	
b_0	< 0	0.011	-inf; 3.459	param. controlling ZNF521 dynamics
b_2	0.224	0.330	-0.373; 0.186	
b_3	0.381	1.165	-0.878; 0.735	
b_4	0.016	0.177	-0.524; 2.944	
e_0	< 0	19.380	-inf; 14.242	param. controlling FLT3 dynamics
f_0	< 0	0.502	-inf; 8.972	param. controlling IL-7R dynamics
f_1	< 0	0.715	-inf; 2.253	
f_2	< 0	22.380	-inf; 6.373	
f_3	< 0	9.267	-inf; 7.069	
j_0	< 0	0.204	-inf; 7.672	param. controlling E2A dynamics
j_1	0.024	0.471	-0.807; 3.487	

* The parameter sensitivity for bistability is defined as the log2 fold change of the ratio between the max (min) parameter value for which the system is bistable and the parameter used in the model (S1 Table).
