# Supplementary material for: Computational Modeling of a Transcriptional Switch Underlying B-Lymphocyte Lineage Commitment of Hematopoietic Multipotent Cells
Source: PLoS One. 2015 Jul 13;10(7):e0132208. doi: 10.1371/journal.pone.0132208 (PMC4500571; doi:10.1371/journal.pone.0132208)
Supplement: S3 Table — Parameters values used as different configurations of the original transcriptional regulatory network, considering reasonable regulatory gene hypotheses. (PDF) [file pone.0132208.s017.pdf]

Table S3. Parameter values used in the model B and C bifurcation studies.

Parameter	Value	Model	Description
b_{e2a}	0.03	B	Inhibition of ZNF521 through E2A
b_{ika}	0.25	C	Inhibition of ZNF521 through IKAROS
b_4	0.0	$ModelB, C$	Removal of ZNF521 inhibition through PAX5

Parameters values used as different configurations of the original transcriptional regulatory network, considering reasonable regulatory gene hypotheses.
